# Privacy-Preserving On-Chain Attestation for Cross-Domain Data Flows via GBFPlus

**DOI:** 10.3390/e28090947

**Published:** 2026-08-23

**Authors:** Sihang Qin, Yang Zhou, Weiqi Dai, Yiming Sun, Weizhong Qiang

**Affiliations:** 1 School of Cyber Science and Engineering, Huazhong University of Science and Technology, Wuhan 430074, China; qinsihang@hust.edu.cn (S.Q.); weiqidai@hust.edu.cn (W.D.); symyiming@hust.edu.cn (Y.S.); wzqiang@hust.edu.cn (W.Q.); 2 Hubei Key Laboratory of Distributed System Security, Huazhong University of Science and Technology, Wuhan 430074, China; 3 Hubei Engineering Research Center on Big Data Security, Huazhong University of Science and Technology, Wuhan 430074, China

**Keywords:** cross-domain data flows, data-flow regulation, privacy-preserving attestation, blockchain, Bloom filters

## Abstract

Cross-domain data flows are commonplace in regulated inter-organizational environments, where durable audit evidence must be retained without publicly exposing sensitive flow metadata. This paper presents a privacy-preserving on-chain attestation framework for recorded cross-domain data transfers in a permissioned setting. Its core data structure, termed GBFPlus, extends the Garbled Bloom Filter (GBF) with explicit occupancy indicators, constrained payloads that encode a consistency prefix and an adjacent-domain identifier, and distinct pairing-derived positions. Each domain administrator records observed inbound and outbound transfers in directional GBFPlus instances and periodically commits signed filter attestations to an append-only ledger. An authorized regulator can reconstruct candidate transfer edges from available bilateral attestations, while light clients verify ledger inclusion through Merkle proofs. A traceable anonymous attestation signature conceals the uploader’s cryptographic identity from ordinary ledger observers while retaining regulator-assisted accountability. The security analysis establishes integrity, conditional anonymity, traceability, and metadata-privacy properties for committed attestations under the stated trust assumptions, and the prototype evaluation reports the measured costs of GBFPlus and the signature operations.

## 1. Introduction

Cross-domain data flows are now routine across numerous digital sectors. These flows are also difficult to audit. A data item may traverse several organizations or administrative domains, the transmission path may be indirect, and the evidence needed for later investigation may be fragmented across independently managed systems. Regulatory frameworks such as the European Union’s General Data Protection Regulation (GDPR) emphasize both data protection and lawful data circulation [1], while international policy efforts such as the OECD Declaration on Government Access to Personal Data Held by Private Sector Entities emphasize safeguards, transparency, and accountability [2]. These developments create a technical requirement: systems should preserve useful audit evidence for cross-domain movement without unnecessarily disclosing sensitive flow metadata.

We focus on a permissioned regulatory setting in which registered organizations operate domain gateways and an authorized regulator audits records after a dispute or compliance request. Ordinary access control enforced at the moment of sharing is not sufficient: once data leaves the original domain, the regulator may need durable evidence of the transfer statements recorded by the participating domains. A direct blockchain record of each transfer would make committed evidence tamper-evident, but it would also expose data identifiers, adjacent-domain relationships, and signer identities to ledger observers. Conversely, keeping all records off-chain weakens later verifiability. The objective of this work is to provide compact on-chain attestation and metadata privacy for recorded transfers.

We propose an on-chain attestation framework based on GBFPlus, an application-oriented extension of the Garbled Bloom Filter (GBF). Each domain administrator (DA) maintains separate incoming and outgoing GBFPlus instances. A GBFPlus entry is addressed by a keyed pairing-derived value tied to the data identifier and the DA key, and its constrained payload encodes the adjacent DA identifier behind a short consistency prefix. Observers who lack the required query material cannot derive the positions for a target flow, while the regulator can reconstruct candidate edges by checking the available outgoing record of the sender and incoming record of the receiver. To avoid revealing which DA uploaded an attestation, we combine these records with a special-purpose traceable anonymous attestation signature mechanism. The regulator and each DA jointly generate an anonymous attestation signing key without disclosing the signing scalar to the regulator; public verifiers can validate registration status, while the regulator can trace accepted signatures when accountability is required.

This paper makes two core contributions:

First, we define a scoped regulatory-audit model for privacy-preserving on-chain attestation of recorded cross-domain data flows and introduce GBFPlus as an extension of GBF. Explicit occupancy indicators, constrained payloads, and distinct pairing-derived positions enable compact direction-specific attestations and controlled recovery of adjacent-domain identifiers rather than public membership testing.

Second, we combine bilateral incoming and outgoing records with an accountable anonymous upload mechanism. Public verifiers can confirm that an attestation originates from a registered DA without learning its cryptographic identity, while the regulator can trace accepted signatures using private tracing records. We analyze integrity, conditional anonymity, traceability, and metadata privacy for committed records and report prototype measurements for GBFPlus and signature operations.

## 2. Related Work

Cryptographic timestamping [3] and append-only ledgers [4] provide a natural basis for tamper-evident records. Blockchains generalize this idea into auditable data structures for integrity, transparency, and decentralized verification [5,6]. They have therefore been applied to electronic evidence, chain-of-custody management, and data provenance [7,8,9]. Recent provenance-oriented systems further strengthen audit trails for data forensics [10], combine trusted execution environments with watermarking for secure data upload and retrieval [11], or systematize blockchain-enabled provenance across dimensions of capture, storage placement, and privacy [12]. These works motivate the use of ledgers for evidence integrity, but they generally store raw provenance metadata or forensic records rather than compact, queryable, privacy-preserving attestations for individual cross-domain transfer edges.

Blockchain-assisted data sharing has been studied across diverse application scenarios, improving secure exchange through combinations of authentication, edge computing, and blockchain coordination [13,14,15,16,17,18]. Separately, blockchain-based authorization and identity mechanisms support user-centric resource sharing [19], decentralized identifiers and verifiable credentials for multi-domain access control [20], and privacy-preserving authentication in IoT environments [21]. Zhao et al. [22] combine alliance-chain trust domains, a public authorization chain, and a conventional BF to accelerate cross-domain traceability. Their stable data-hash index and explicit domain, blockchain, and user identifiers facilitate authorization and graph reconstruction, but expose linkable metadata to ledger participants.

Classical group signatures provide public verification, signer anonymity, and an opening authority for accountability [23]; pairing-based constructions further reduce signature size [24]. Traceable and revocable data-sharing schemes address malicious user behavior and post-hoc accountability in blockchain-enabled environments [25,26]. Privacy-oriented designs aim to reduce information leakage during cross-domain data exchange [27], and work on data markets and federated provenance explores fairness, verifiable credentials, accountable access control, and provenance assurance [28,29,30,31]. These systems typically bind accountability to transaction semantics, credentials, or application-specific evidence models. Our signing mechanism follows the accountable-anonymity paradigm but is specialized for randomized attestation keys and regulator-held tracing records, rather than a full group-signature scheme with dynamic membership. The overall design requires each traversed domain to publish compact filter-based attestations that hide data identifiers and inter-domain relationships from external observers while remaining queryable by authorized parties.

Fine-grained access control for encrypted records is commonly built from attribute-based encryption [32,33]. Existing systems demonstrate how anonymous credentials, attribute-based encryption, and data ownership structures can protect data usage in specific sharing scenarios [34,35,36]. Bloom filters and garbled Bloom filters provide compact set representations and have been widely adopted in privacy-preserving protocols [37,38,39].

Succinct non-interactive arguments, such as Groth16 [40], and transparent proof systems, such as STARKs [41], can establish the correctness of general circuit statements. Their role differs from the controlled-search structure required here: a proof can validate a specified statement, whereas GBFPlus aggregates transfer observations and supports authorized recovery of candidate adjacent-domain records. General proof systems could complement the attestation workflow, but they do not by themselves provide its compact searchable representation.

Overall, prior work usually protects the act of sharing, authorizing, trading, or storing provenance metadata. The distinction of this work is narrower and should not be read as a replacement for those mechanisms: it studies how each traversed domain can publish compact evidence of an observed transfer edge while hiding the data identifier, the adjacent domain, and the signer’s public identity from ordinary ledger observers. The resulting design combines ledger inclusion, GBFPlus-based candidate-edge reconstruction, and regulator-held tracing records; its contribution lies in this privacy-oriented attestation composition rather than in proposing a new general-purpose group signature, access-control primitive, or complete blockchain system. Table 1 summarizes the core functional and privacy distinctions.

## 3. Preliminaries

### 3.1. Blockchain and SPV

**Figure 1:**
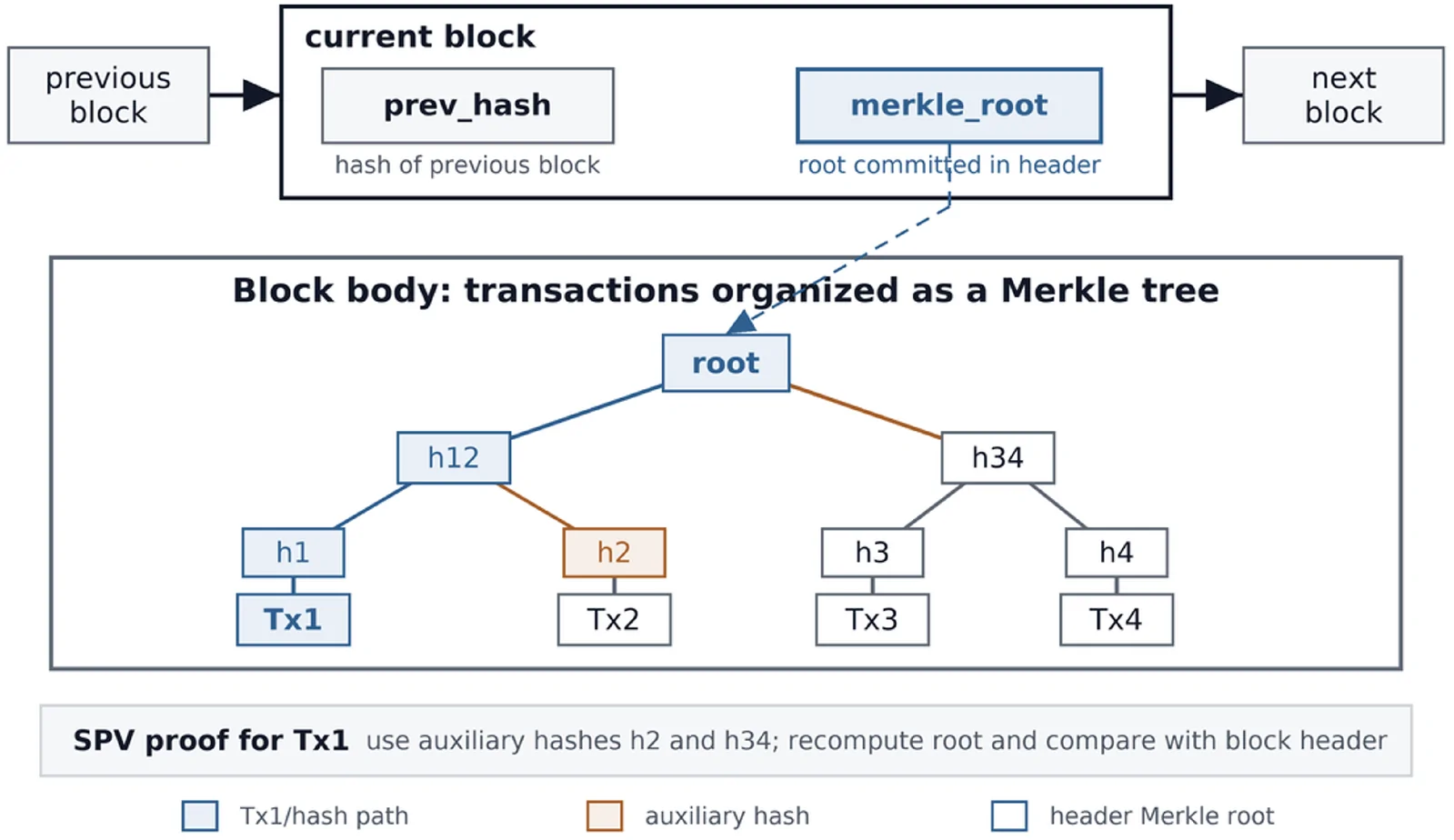
Block structure and Simplified Payment Verification (SPV) Merkle authentication path.

We use the blockchain as an append-only, replicated log for attestation transactions. The construction does not require a particular consensus protocol or smart-contract language; it requires that accepted blocks eventually become persistent and that participants agree on block headers and Merkle roots except with negligible ledger-failure probability. As illustrated in Figure 1, each block contains a header and a body. The header stores PreH, the cryptographic hash of the previous block, which links this block to its predecessor and underpins the immutability of the chain. It also contains root, the Merkle root of all transactions in the block body; this single hash serves as a compact, tamper-evident summary that allows efficient verification of whether a given transaction is included in the block. The body, on the other hand, stores the transactions themselves and usually dominates the storage cost.

A node that stores only block headers is called a light client. Such a client can use Simplified Payment Verification (SPV) [4] to verify transaction inclusion against stored block headers. To prove that ${\mathrm{Tx}}_{1}$ is included in the blockchain, a prover need not provide the whole block body; it suffices to provide ${\mathrm{Tx}}_{1}$ and its Merkle authentication path, also called a Merkle branch in Bitcoin terminology. The path consists of the sibling hashes and their left/right positions; in Figure 1, these sibling hashes are $\left(h_{2},h_{34}\right)$. Let $H_{M}$ denote the Merkle-tree hash function. The light client reconstructs ${\mathrm{root}}^{\prime }=H_{M}\left(H_{M}\left(H_{M}\left({\mathrm{Tx}}_{1}\right)∥h_{2}\right)∥h_{34}\right)$ and compares it with the locally stored root. Equality verifies inclusion in the corresponding block, but it does not by itself prove that the transaction contents are truthful; content validity follows from the cryptographic checks and operational assumptions stated below.

### 3.2. Asymmetric Bilinear Groups

Let $\left(G_{1},+\right)$, $\left(G_{2},+\right)$, and $\left(G_{T},\cdot \right)$ be cyclic groups of the same large prime order *q*, where $G_{1}\neq G_{2}$ and no efficiently computable isomorphism exists between $G_{1}$ and $G_{2}$. Let $Q\in G_{1}$ and $P\in G_{2}$ be generators. A map $\hat{e}:G_{1}\times G_{2}\to G_{T}$ is an asymmetric (Type-3) bilinear pairing if it satisfies the following properties [42]:

- Bilinearity: For all $a,b\in Z_{q}$, $X\in G_{1}$, and $Y\in G_{2}$, $\hat{e}\left(aX,bY\right)=\hat{e}\left(abX,Y\right)=\hat{e}\left(X,abY\right)=\hat{e}{\left(X,Y\right)}^{ab}$.
- Non-degeneracy: $\hat{e}\left(Q,P\right)\neq 1_{G_{T}}$.
- Efficient computability: There exists an efficient algorithm to compute $\hat{e}\left(X,Y\right)$ for any $X\in G_{1}$ and $Y\in G_{2}$.

We rely on several standard cryptographic assumptions within these groups. In a cyclic group G of order *q* with generator *G*, the Discrete Logarithm Problem (DLP) is to find $a\in Z_{q}$ given (G,aG). The Decisional Diffie-Hellman (DDH) problem is to distinguish (G,aG,bG,abG) from (G,aG,bG,cG) for randomly chosen $a,b,c\in Z_{q}$. In the Type-3 asymmetric setting, we rely on the Symmetric External Diffie–Hellman (SXDH) assumption, which states that the DDH problem is intractable in both $G_{1}$ and $G_{2}$. We distinguish the two directional co-CDH assumptions required below: given $\left(Q,aQ\right)\in G_{1}^{2}$ and $Y\in G_{2}$, computing $aY\in G_{2}$ is infeasible; symmetrically, given $\left(P,aP\right)\in G_{2}^{2}$ and $X\in G_{1}$, computing $aX\in G_{1}$ is infeasible. We refer to the applicable direction explicitly whenever it is used.

### 3.3. Filters

A Bloom filter (BF) [37] is a compact probabilistic data structure for set membership. A classical BF is a one-dimensional array *A* whose entries are initially 0. For each element x∈S, predefined hash functions $\left(H_{1},H_{2},\ldots ,H_{\kappa }\right)$ map *x* to κ positions, and those positions are set to 1; that is, $A[H_{i}\left(x\right)]=1$ for i=1,…,κ. A query reports membership if all mapped positions are 1, and reports nonmembership otherwise.

**Figure 2:**
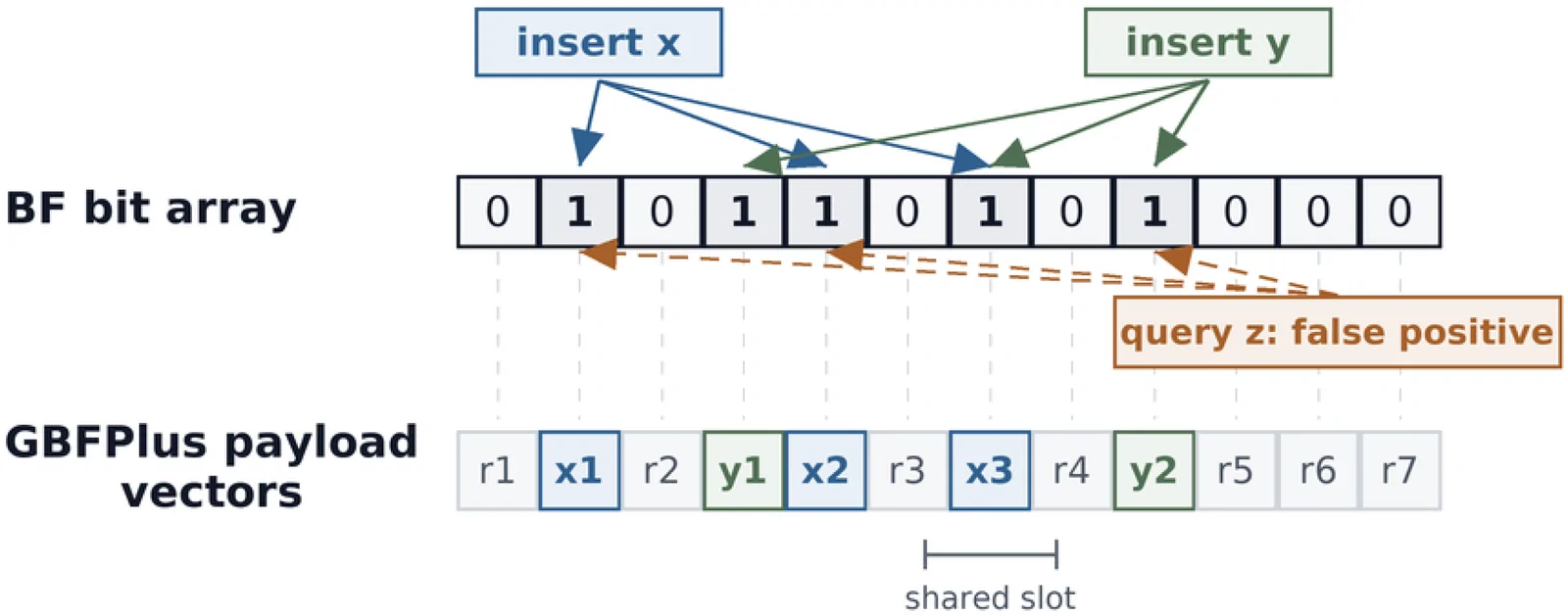
Bloom filters and garbled Bloom filters.

Bloom filters may return false positives but, without deletions, do not return false negatives. In Figure 2, z∉S, yet all positions mapped by *z* are already 1, so *z* is falsely classified as a member of S. The false-positive rate can be reduced to an acceptable level by selecting parameters according to the insertion bound η.

Filters are also useful in cryptographic protocols. Dong et al. [38] proposed garbled Bloom filters (GBFs), in which each filter entry is a λ-bit vector. For illustration, consider three mapped positions: vectors $x_{1},x_{2},x_{3}$ are sampled subject to $x_{1}⊕x_{2}⊕x_{3}=x$. If a mapped position has already been used, its existing vector is reused. In Figure 2, element *y* is inserted after *x*; it shares $x_{3}$ with *x*, and vectors $y_{1},y_{2}$ are sampled so that $y_{1}⊕y_{2}⊕x_{3}=y$. After all elements are inserted, the remaining positions are filled with random vectors $r_{i}$. For a nonmember *z*, the probability that $x_{1}⊕x_{2}⊕y_{2}=z$ is $2^{-\lambda }$, so the GBF false-positive probability depends on λ. GBFPlus extends this construction for the considered attestation task.

### 3.4. Paillier Homomorphic Encryption

Paillier encryption [43] is a probabilistic public-key encryption scheme with additive homomorphism. Let ${\mathrm{Enc}}_{\mathrm{pk}}\left(m\right)$ denote the encryption of a message *m* under public key pk (with the encryption randomness omitted for brevity), and let ${\mathrm{Dec}}_{\mathrm{sk}}\left(c\right)$ denote the decryption of ciphertext *c* using the corresponding secret key sk. For plaintext modulus M, plaintexts $m_{1},m_{2}$, and scalar *k*, with all plaintext operations performed modulo M, Paillier encryption satisfies the following properties:

(1) $$\begin{array}{cc} {\mathrm{Enc}}_{\mathrm{pk}}\left(m_{1}\right)\cdot {\mathrm{Enc}}_{\mathrm{pk}}\left(m_{2}\right) & ={\mathrm{Enc}}_{\mathrm{pk}}\left(m_{1}+m_{2}\right), \end{array}$$

(2) $$\begin{array}{cc} {\mathrm{Enc}}_{\mathrm{pk}}{\left(m_{1}\right)}^{k} & ={\mathrm{Enc}}_{\mathrm{pk}}\left(km_{1}\right). \end{array}$$

## 4. System Model and Design Goals

### 4.1. System Model

**Figure 3:**
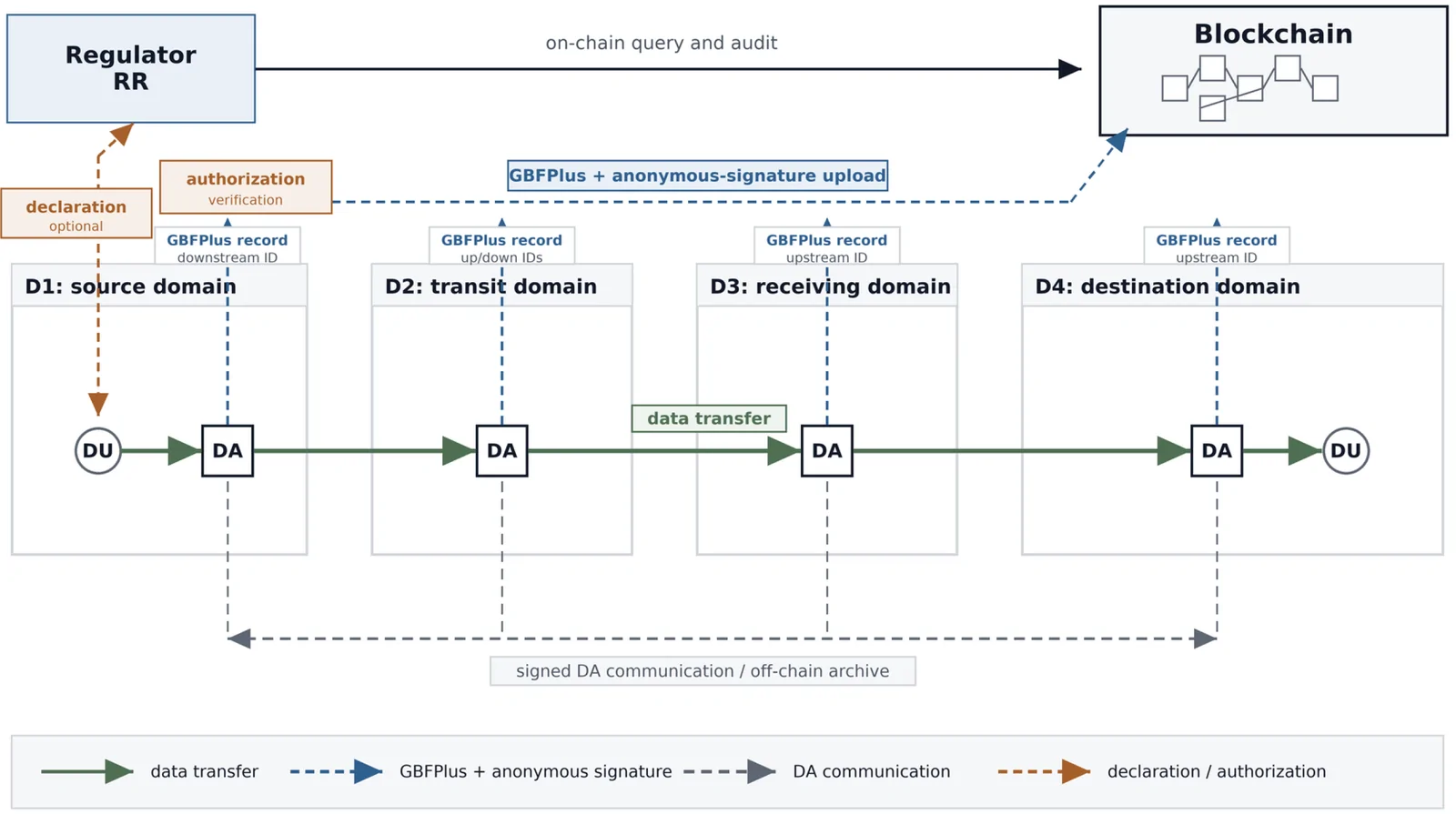
System model and workflow.

The proposed system tracks recorded cross-domain data flows through an attestation layer that begins when a DA observes a transfer and ends when the corresponding filter attestation is committed to the ledger. As shown in Figure 3, the architecture comprises four core entities:

- Regulator (RR): A trusted authority responsible for policy enforcement, auditing, and cryptographic key management. RR issues domain identifiers, generates components for anonymous attestation, authorizes data-specific queries, and executes signature tracing when administrative accountability is required.
- Domain Administrator (DA): Registered gateway nodes operated by participating organizations. DAs observe ingress and egress events in their respective domains, record them in directional GBFPlus instances, and periodically upload signed filter attestations to the ledger.
- Data User (DU): An entity that transfers or monitors data across domains. A DU can act as a light client that verifies Merkle proofs returned by RR or, after authorization, as a full node that queries on-chain records using data-specific query material.
- Ledger: An append-only consortium or permissioned blockchain maintained by the RR and participating domains. It ensures the persistence, strict ordering, and consensus-backed integrity of finalized attestation transactions.

The system targets high-value or immutable data objects (e.g., personal information, medical images, or legal documents) whose recorded inter-domain movements may later be audited for compliance. Each data item is assumed to possess a persistent unique identifier string ${\mathrm{ID}}_{m}$. The ledger attests what registered DAs have recorded; it does not by itself prove that every physical transfer was observed or logged.

### 4.2. Design Goals

To support secure and controlled data-flow auditing, the system targets the following design goals:

- Tamper-Evident Flow Attestation: Once a filter attestation is committed to the ledger, any subsequent modification or deletion of that committed evidence must be detectable.
- Accountable Anonymity: Public observers must be able to verify that an uploaded attestation originates from a legitimately registered DA without revealing the specific identity of that DA, while allowing RR to uniquely trace the signer under compliance mandates.
- Privacy of Flow Metadata: GBFPlus payloads must conceal the underlying data identifiers (${\mathrm{ID}}_{m}$), query positions, and adjacent-domain relationships from ordinary ledger observers, unauthorized users, and uninvolved DAs.
- Controlled Query and Verification: Provenance reconstruction must be authorized; only RR or explicitly authorized users holding valid data-specific query material can inspect on-chain transfer paths.
- Compactness and Efficiency: The attestation records should maintain a compact storage footprint, and the costs of insertion, query, and signature verification should remain moderate in the evaluated prototype.

### 4.3. Adversarial Model and Security Requirements

The security model considers ordinary ledger observers and PPT external adversaries that can inspect committed transactions, request signatures and authorized nonchallenge queries, and corrupt selected DAs. A corruption reveals that DA’s secret state and excludes it from an honest-party challenge. Registered DAs follow the insertion and submission protocols but may be curious about regulator queries; RR is trusted to authorize queries and protect its tracing records. All algorithms reject malformed encodings, identity elements, and elements outside their prescribed groups. Consensus attacks and collusion among registered parties are outside the cryptographic scope of this work.

The cryptographic requirements are formalized by the following games, each run after system initialization and honest DA registration:

- Integrity and Authenticity ($G_{\mathrm{att}-\mathrm{UF}}$): The adversary receives the public parameters and public DA records, may obtain signatures from honest DAs, and may corrupt selected DAs. It wins by outputting a fresh pair $\left(M^{*},\varsigma^{*}\right)$ that passes Algorithm 1, traces to an uncorrupted DA, and was not returned by that DA’s signing oracle.
- Conditional Anonymity ($G_{\mathrm{anon}}$): The adversary selects two uncorrupted registered DAs and a message with equal public ledger metadata. The challenger signs with one of them according to a hidden random bit. The adversary, without either signing secret, the corresponding private registration values $B_{i},V_{i}$, or RR’s tracing records, wins by guessing the bit; its advantage is $\left|\mathrm{Pr}[b^{\prime }=b]-1/2\right|$.
- Traceability ($G_{\mathrm{trace}}$): The adversary may register and corrupt DAs and obtain signatures. It wins if an accepted signature associated with an honest registration has no matching tracing record, matches more than one honest record, or is attributed to an uncorrupted DA different from its actual signer. RR’s tracing database is assumed intact.
- Query Confidentiality ($G_{\mathrm{qpriv}}$): The adversary selects two identifiers with equal public filter configuration and ledger-visible metadata and may adaptively request authorized views for other identifiers. The challenger returns the ledger view for one challenge identifier and, when an interactive processing DA is modeled, a request blinded with a fresh scalar. Neither challenge identifier may have a previously disclosed unblinded authorization token. The adversary wins by identifying the selected identifier; repeated challenge interactions use independent blinding scalars.

**Algorithm 1** Traceable anonymous attestation signature verification**Input:** Public parameters PP; message *M*; candidate anonymous attestation signature ς
**Output:** Acceptance bit True or False
1:Parse ς as $\left(\sigma ,{\mathrm{dPK}}^{*},Y^{*},R^{*},B^{*},\chi^{*}\right)$, where $\chi^{*}=\left(C^{*},z^{*}\right)$, $\sigma ,Y^{*}\in G_{2}$, ${\mathrm{dPK}}^{*},R^{*},B^{*},C^{*}\in G_{1}$, and $z^{*}\in Z_{q}$.2:**if** any group element is outside its expected group or is the identity element, or if $z^{*}\notin Z_{q}$ **then**3:    **return** False.4:**end if**5:Set $h_{M}\leftarrow H_{3}\left(M\right)$.6:Set$$c^{*}\leftarrow H_{4}\left(\mathrm{anon}-r∥\langle M,\sigma ,{\mathrm{dPK}}^{*},Y^{*},R^{*},B^{*},C^{*}\rangle \right).$$7:**if** $z^{*}Q\neq C^{*}+c^{*}R^{*}$ **then**8:    **return** False.9:**end if**10:**if** $\hat{e}\left(Q,\sigma \right)\neq \hat{e}\left({\mathrm{dPK}}^{*},h_{M}\right)$ **then**11:    **return** False.12:**end if**13:Set ${\mathrm{ok}}_{1}\leftarrow \left[\hat{e}\left(Q,Y^{*}\right)=\hat{e}\left(R^{*},{\mathrm{MPK}}_{2}\right)\right]$.14:Set ${\mathrm{ok}}_{2}\leftarrow \left[\hat{e}\left({\mathrm{dPK}}^{*},P\right)=\hat{e}\left({\mathrm{MPK}}_{1},Y^{*}\right)\cdot \hat{e}\left(B^{*},{\mathrm{MPK}}_{2}\right)\right]$.15:**if** ${\mathrm{ok}}_{1}$ and ${\mathrm{ok}}_{2}$ **then**16:    **return** True.17:**else**18:    **return** False.19:**end if**


Let the corresponding advantages be ${\mathrm{Adv}}_{A}^{\mathrm{att}-\mathrm{UF}}$, ${\mathrm{Adv}}_{A}^{\mathrm{anon}}$, ${\mathrm{Adv}}_{A}^{\mathrm{trace}}$, and ${\mathrm{Adv}}_{A}^{\mathrm{qpriv}}$. The construction satisfies the four requirements when these advantages are negligible for every PPT adversary under the stated trust and leakage conditions. Ledger persistence and inclusion proofs separately provide tamper evidence for accepted transactions.

## 5. Proposed Scheme

### 5.1. System Initialization

RR first generates the global public parameters. It selects groups $G_{1},G_{2},G_{T}$ equipped with a bilinear map $\hat{e}:G_{1}\times G_{2}\to G_{T}$, where all groups share a large prime order *q*, and defines $Z_{q}^{*}=Z_{q}\setminus \left\{0\right\}$. It specifies a cryptographic hash function $H_{1}:{\left\{0,1\right\}}^{*}\to {\left\{0,1\right\}}^{L_{H}}$. For any serializable value or tuple *x*, let 〈x〉 denote its canonical encoding. For $u\in G_{T}$, let $H_{\mathrm{idx}}\left(u\right)=H_{1}\left(\mathrm{idx}∥\langle u\rangle \right)$ and $H_{\mathrm{pay}}\left(u\right)=H_{1}\left(\mathrm{pay}∥\langle u\rangle \right)$ denote domain-separated hashes, where $L_{H}\geq \lambda$. RR also defines domain-separated hash-to-curve maps $H_{2}:{\left\{0,1\right\}}^{*}\to G_{2}\setminus \left\{0_{G_{2}}\right\}$ and $H_{3}:{\left\{0,1\right\}}^{*}\to G_{2}\setminus \left\{0_{G_{2}}\right\}$, together with a scalar hash $H_{4}:{\left\{0,1\right\}}^{*}\to Z_{q}$. RR configures the DA identifier length $l_{\mathrm{DA}}$ and the GBFPlus parameters κ, η, $\hat{\eta }$, and λ, satisfying $0<l_{\mathrm{DA}}<\lambda$. It selects position-mapping functions $H_{i}:{\left\{0,1\right\}}^{*}\to Z_{\hat{\eta }}$ for i=1,…,κ, where κ is the number of mappings, η is the maximum supported successful insertions per filter, $\hat{\eta }$ is the size of the position space, and λ is the payload bit length. We use ‖ for concatenation, ⊥ for failure, and ${\mathrm{Trunc}}_{\ell }\left(x\right)$ for fixed *ℓ*-bit truncation. Let ${\mathrm{Idx}}_{\kappa ,\hat{\eta }}\left(x\right)$ denote the public deterministic procedure that initializes an empty list and a counter ctr=0, evaluates $H_{i}\left(x∥\mathrm{ctr}\right)$ for i=1,…,κ, appends only new indices, increments ctr, and terminates once it has accumulated κ distinct indices in $Z_{\hat{\eta }}$.

RR samples master secrets $s_{1},s_{2}\overset{\$}{\leftarrow }Z_{q}^{*}$, sets $\mathrm{MSK}=(s_{1},s_{2})$, and computes master public keys ${\mathrm{MPK}}_{1}=s_{1}Q\in G_{1}$ and ${\mathrm{MPK}}_{2}=s_{2}P\in G_{2}$. The public parameters are published as follows:

(3) $$\begin{array}{cc} \mathrm{PP}=\{ & \hat{e},G_{1},G_{2},G_{T},q,Q,P,{\mathrm{MPK}}_{1},{\mathrm{MPK}}_{2},H_{1},H_{2},H_{3},H_{4},\\ \; & H_{\mathrm{idx}},H_{\mathrm{pay}},{\mathrm{Idx}}_{\kappa ,\hat{\eta }},l_{\mathrm{DA}},\kappa ,\eta ,\hat{\eta },\lambda ,H_{1},\ldots ,H_{\kappa }\}. \end{array}$$

For *n* DAs ($n\leq 2^{l_{\mathrm{DA}}}$), RR assigns each ${\mathrm{DA}}_{i}$ a unique identifier $N_{i}\in {\left\{0,1\right\}}^{l_{\mathrm{DA}}}$ within the public set N. Each ${\mathrm{DA}}_{i}$ samples a long-term transfer signing and query secret $k_{i}\overset{\$}{\leftarrow }Z_{q}^{*}$ and sets ${\mathrm{KPK}}_{i}=k_{i}Q\in G_{1}$ as its verification key. This setup supports standard BLS signatures [44]: for message *M*, the signature is $\sigma_{i}\left(M\right)=k_{i}H_{3}\left(M\right)\in G_{2}$, verified by checking $\hat{e}\left(Q,\sigma_{i}\left(M\right)\right)=\hat{e}\left({\mathrm{KPK}}_{i},H_{3}\left(M\right)\right)$. DAs also initialize a validated Paillier key pair $\left({\mathrm{PuK}}_{i},{\mathrm{PrK}}_{i}\right)$ and publish ${\mathrm{PuK}}_{i}$. The associations between ${\mathrm{DA}}_{i}$, ${\mathrm{KPK}}_{i}$, $N_{i}$, and the managed domain $D_{i}$ are public. The distinct symbols $k_{i}$ and $d_{i}$ are used respectively for the long-term transfer/query key and the anonymous on-chain attestation key introduced below.

${\mathrm{DA}}_{i}$ executes Algorithm 2 with RR to obtain an anonymous attestation signing key $d_{i}\in Z_{q}^{*}$ without escrow. The provisional key ${\tilde{d}}_{i}$ is protected by the one-time delayed-release mask $\nu_{i}$: RR releases $\nu_{i}$ only after the pairing check binds the Paillier plaintext to $X_{i}$. RR stores the verification element $V_{i}=d_{i}Q$ and tracing record $\left(T_{i},Z_{i},{\mathrm{DA}}_{i}\right)$, but cannot recover the scalar $d_{i}$ under the discrete-logarithm assumption. With $d_{i}$, base token $B_{i}$, and Algorithm 3, ${\mathrm{DA}}_{i}$ generates anonymous attestation signatures $\varsigma_{i,j}$ bound to randomized one-time keys.
**Algorithm 2** Validated anonymous attestation signing key generation**Input:** ${\mathrm{DA}}_{i}$’s validated Paillier key pair $\left({\mathrm{PuK}}_{i},{\mathrm{PrK}}_{i}\right)$, with only ${\mathrm{PuK}}_{i}$ given to RR, and plaintext modulus M; RR’s master secret key $\mathrm{MSK}=(s_{1},s_{2})$; public parameters PP, including $\hat{e},P,Q,q,{\mathrm{MPK}}_{1},{\mathrm{MPK}}_{2}$; mask bound $R_{\rho }$ satisfying $q^{2}+R_{\rho }q<M$
**Output:** RR obtains the verification element $V_{i}=d_{i}Q$ and stores the tracing record $\left(T_{i},Z_{i},{\mathrm{DA}}_{i}\right)$; after a successful run, ${\mathrm{DA}}_{i}$ obtains an anonymous attestation signing secret key $d_{i}=\left(s_{1}s_{2}+s_{2}\tau_{i}b_{i}\right)\;\mathrm{mod}\;q\in Z_{q}^{*}$ and tracing tag $B_{i}=\tau_{i}b_{i}Q$
1:${\mathrm{DA}}_{i}$ samples $a_{i},b_{i}\overset{\$}{\leftarrow }Z_{q}^{*}$, sets $X_{i}=b_{i}Q$, $Z_{i}=a_{i}P$, $W_{i}=a_{i}b_{i}P$, and $c_{i,1}={\mathrm{Enc}}_{{\mathrm{PuK}}_{i}}\left(b_{i}\right)$.2:${\mathrm{DA}}_{i}$ sends $\left(c_{i,1},X_{i},W_{i},Z_{i}\right)$ to RR.3:RR checks that $c_{i,1}$ is in the Paillier ciphertext group, that $X_{i}\in G_{1}\setminus \left\{0_{G_{1}}\right\}$ and $W_{i},Z_{i}\in G_{2}\setminus \left\{0_{G_{2}}\right\}$, and that $\hat{e}\left(Q,W_{i}\right)=\hat{e}\left(X_{i},Z_{i}\right)$. If any check fails, RR rejects the enrollment.4:RR samples $\tau_{i},\nu_{i}\overset{\$}{\leftarrow }Z_{q}^{*}$ and $\rho_{i}\overset{\$}{\leftarrow }\left\{0,\ldots ,R_{\rho }-1\right\}$, sets $\alpha_{i}=\left(s_{2}\tau_{i}\right)\;\mathrm{mod}\;q$ and $\beta =(s_{1}s_{2})\;\mathrm{mod}\;q$, and computes$c_{i,2}={\mathrm{Enc}}_{{\mathrm{PuK}}_{i}}\left(\beta +\nu_{i}+\rho_{i}q\right)\cdot c_{i,1}^{\alpha_{i}}={\mathrm{Enc}}_{{\mathrm{PuK}}_{i}}\left(\beta +\nu_{i}+\alpha_{i}b_{i}+\rho_{i}q\right)$.5:RR sends $c_{i,2}$ to ${\mathrm{DA}}_{i}$.6:${\mathrm{DA}}_{i}$ decrypts ${\tilde{\mu }}_{i}={\mathrm{Dec}}_{{\mathrm{PrK}}_{i}}\left(c_{i,2}\right)$, sets ${\tilde{d}}_{i}={\tilde{\mu }}_{i}\;\mathrm{mod}\;q$, and sends ${\tilde{A}}_{i}=a_{i}{\tilde{d}}_{i}Q$ to RR.7:RR computes ${\tilde{V}}_{i}=\beta Q+\alpha_{i}X_{i}+\nu_{i}Q$ and checks$\hat{e}\left({\tilde{V}}_{i},Z_{i}\right)=\hat{e}\left({\tilde{A}}_{i},P\right)$.8:**if** the check succeeds **then**9:    RR sets $V_{i}={\tilde{V}}_{i}-\nu_{i}Q$, $B_{i}=\tau_{i}X_{i}$, and $T_{i}=\tau_{i}W_{i}$, and sends $\left(B_{i},\nu_{i}\right)$ to ${\mathrm{DA}}_{i}$.10:**else**11:    RR withholds $\nu_{i}$, rejects the enrollment, and may administratively block repeated invalid requests.12:**end if**13:After receiving $\left(B_{i},\nu_{i}\right)$, ${\mathrm{DA}}_{i}$ sets $d_{i}=\left({\tilde{d}}_{i}-\nu_{i}\right)\;\mathrm{mod}\;q$ and verifies$d_{i}\neq 0$ and $\hat{e}\left(d_{i}Q,P\right)=\hat{e}\left({\mathrm{MPK}}_{1}+B_{i},{\mathrm{MPK}}_{2}\right)$.14:If the check succeeds, ${\mathrm{DA}}_{i}$ stores $\left(d_{i},B_{i}\right)$ and confirms acceptance, after which RR stores $\left(V_{i},T_{i},Z_{i},{\mathrm{DA}}_{i}\right)$; otherwise, both parties discard the provisional state and rerun the protocol.


**Algorithm 3** Traceable anonymous attestation signing**Input:** ${\mathrm{DA}}_{i}$’s anonymous attestation signing secret key $d_{i}$; tracing tag $B_{i}=\tau_{i}b_{i}Q$; public parameters PP; message *M* **Output:** Anonymous attestation signature $\varsigma_{i,j}$ on *M*
1:Sample fresh one-time randomizer $r_{j}\overset{\$}{\leftarrow }Z_{q}^{*}$ and set $h_{M}\leftarrow H_{3}\left(M\right)$.2:Set ${\mathrm{dSK}}_{i,j}\leftarrow \left(r_{j}d_{i}\right)\;\mathrm{mod}\;q$ and ${\mathrm{dPK}}_{i,j}\leftarrow {\mathrm{dSK}}_{i,j}Q$.3:Compute the BLS-type signature $\sigma_{i,j}\leftarrow {\mathrm{dSK}}_{i,j}h_{M}\in G_{2}$.4:Set the ordered registration-verification tuple $\left(Y_{i,j},R_{i,j},B_{i,j}\right)\leftarrow \left(r_{j}{\mathrm{MPK}}_{2},r_{j}Q,r_{j}B_{i}\right)$.5:Sample $\omega_{i,j}\overset{\$}{\leftarrow }Z_{q}^{*}$ and set the commitment $C_{i,j}\leftarrow \omega_{i,j}Q$.6:Compute$$c_{i,j}\leftarrow H_{4}\left(\mathrm{anon}-r∥\langle M,\sigma_{i,j},{\mathrm{dPK}}_{i,j},Y_{i,j},R_{i,j},B_{i,j},C_{i,j}\rangle \right).$$7:Set $z_{i,j}\leftarrow \left(\omega_{i,j}+c_{i,j}r_{j}\right)\;\mathrm{mod}\;q$ and $\chi_{i,j}\leftarrow \left(C_{i,j},z_{i,j}\right)$.8:**return** $\varsigma_{i,j}=\left(\sigma_{i,j},{\mathrm{dPK}}_{i,j},Y_{i,j},R_{i,j},B_{i,j},\chi_{i,j}\right)$.


Algorithms 1 and 3 are algebraically consistent. Honest signatures yield $R_{i,j}=r_{j}Q$, ${\mathrm{dPK}}_{i,j}=r_{j}d_{i}Q$, and $\sigma_{i,j}=r_{j}d_{i}H_{3}\left(M\right)$, so the BLS-type verification relation holds. The verification equations check that $Y_{i,j}$ uses the same randomizer and that ${\mathrm{dPK}}_{i,j}$ structurally aligns with ${\mathrm{MPK}}_{1}$, ${\mathrm{MPK}}_{2}$, and $B_{i,j}$, as configured during Algorithm 2.

For an accepted anonymous signature $\varsigma =(\sigma ,{\mathrm{dPK}}^{*},Y^{*},R^{*},B^{*},\chi^{*})$, RR traces the signer by scanning its database $\left(T_{i},Z_{i},{\mathrm{DA}}_{i}\right)$ for i=1,…,n. If the following holds:

(4) $$\hat{e}\left(B^{*},Z_{i}\right)=\hat{e}\left(R^{*},T_{i}\right),$$

RR attributes the signature to ${\mathrm{DA}}_{i}$; otherwise, it outputs ⊥. Because a valid registration satisfies $Z_{i}=a_{i}P$ and $T_{i}=a_{i}\tau_{i}b_{i}P$, Equation (4) holds precisely when $B^{*}$ is derived from $B_{i}=\tau_{i}b_{i}Q$ using the scalar represented by $R^{*}$.

### 5.2. Attestation of Cross-Domain Data Flows

When ${\mathrm{DU}}_{\mathrm{se}}$ transmits data item *m* to ${\mathrm{DU}}_{\mathrm{re}}$ across domains managed by ${\mathrm{DA}}_{\mathrm{se}}$ and ${\mathrm{DA}}_{\mathrm{re}}$, the administrators observe the transfer and the associated metadata info. This attestation protocol records only the domain-level edge topology and leaves the raw data content off-chain.

Following observation, ${\mathrm{DA}}_{\mathrm{se}}$ computes $\Lambda_{m}=H_{2}\left({\mathrm{ID}}_{m}\right)\in G_{2}$, generates the BLS signature $\sigma_{\mathrm{se}}^{\mathrm{out}}=\sigma_{\mathrm{se}}(\Lambda_{m}∥\mathrm{info}∥N_{\mathrm{re}})$, and forwards it to ${\mathrm{DA}}_{\mathrm{re}}$, who validates it under ${\mathrm{KPK}}_{\mathrm{se}}$. The sender logs the event into its current outgoing GBFPlus instance.

GBFPlus extends GBF for the attestation setting by attaching an occupancy bit to each λ-bit payload cell and constraining the reconstructed payload to a consistency prefix and an adjacent-domain identifier. To neutralize XOR cancellation risks from index collisions, insertion and query procedures call ${\mathrm{Idx}}_{\kappa ,\hat{\eta }}$ to derive κ distinct positions from a domain-separated seed: $H_{\mathrm{idx}}\left(u\right)$ for insertion and $H_{\mathrm{idx}}\left(u^{\prime }\right)$ for query. For outgoing records, Algorithm 4 sets $N_{\mathrm{adj}}=N_{\mathrm{re}}$; for incoming records, it sets $N_{\mathrm{adj}}=N_{\mathrm{se}}$.

Algorithm 4 ensures that the XOR sum of the λ-bit payloads across the κ selected positions matches target. Pre-occupied positions contribute their existing data, empty positions receive fresh random shares, and the final index absorbs the residual share. If all target positions are already occupied (tag=−1), the insertion fails without modifying the current instance. This event triggers filter finalization and rotation: ${\mathrm{DA}}_{i}$ signs and submits the current instance, initializes a new instance with an incremented sequence number, and retries the same failed record before processing subsequent events. The same finalization procedure is invoked after the successful-insertion count reaches η or the configured time window ends.

Let dir∈{in,out}, win be the time-window identifier, seq the filter sequence number, *c* the successful-insertion count, $h_{F}=H_{M}\left(\langle \mathrm{GBFPlus}\rangle \right)$ the canonical filter digest, $h_{\mathrm{prev}}$ the preceding finalized-filter digest, and nonce an idempotent submission identifier. The anonymous attestation signature is generated over the canonical message

(5) $$M_{\mathrm{att}}=\langle \text{𝚐𝚋𝚏𝚙-𝚊𝚝𝚝},\mathrm{dir},\mathrm{win},\mathrm{seq},c,h_{F},h_{\mathrm{prev}},\mathrm{nonce}\rangle .$$

The corresponding ledger transaction contains these fields, the finalized GBFPlus instance, and the anonymous signature on $M_{\mathrm{att}}$. A locally persisted pending-event record allows an interrupted submission to resume, while $\left(\mathrm{dir},\mathrm{win},\mathrm{seq},h_{F},\mathrm{nonce}\right)$ gives the ledger a deterministic deduplication key. Consequently, retransmission can preserve the same logical attestation without creating a second accepted filter instance, and the record that triggered a rotation remains pending until it is inserted into the new instance.

The receiver ${\mathrm{DA}}_{\mathrm{re}}$ validates $\sigma_{\mathrm{se}}^{\mathrm{out}}$, generates an acknowledgment signature $\sigma_{\mathrm{re}}^{\mathrm{in}}=\sigma_{\mathrm{re}}(\Lambda_{m}∥\mathrm{info}∥N_{\mathrm{se}})$, and returns it to ${\mathrm{DA}}_{\mathrm{se}}$. It then executes Algorithm 4 on its incoming GBFPlus instance with $N_{\mathrm{adj}}=N_{\mathrm{se}}$ under identical rotation rules. The core signatures $\sigma_{\mathrm{se}}^{\mathrm{out}}$ and $\sigma_{\mathrm{re}}^{\mathrm{in}}$ are preserved off-chain for independent audit and are associated with the corresponding filter sequence and ledger transaction identifier.
**Algorithm 4** GBFPlus insertion**Input:** $\left(\kappa ,\eta ,\hat{\eta },\lambda \right)$-GBFPlus with successful-insertion count c<η; group-encoded data identifier $\Lambda_{m}$; long-term DA transfer/query secret key $k_{i}$; RR master public-key component ${\mathrm{MPK}}_{1}$; assigned adjacent DA identifier $N_{\mathrm{adj}}\in N\subseteq {\left\{0,1\right\}}^{l_{\mathrm{DA}}}$; identifier length $l_{\mathrm{DA}}$; derived hashes $H_{\mathrm{idx}},H_{\mathrm{pay}}$; index procedure ${\mathrm{Idx}}_{\kappa ,\hat{\eta }}$ **Output:** Updated GBFPlus with count c+1, or failure symbol ⊥
1:Initialize tag←−1.2:$u\leftarrow \hat{e}\left(k_{i}{\mathrm{MPK}}_{1},\Lambda_{m}\right)$.3:$\mathrm{target}\leftarrow {\mathrm{Trunc}}_{\lambda -l_{\mathrm{DA}}}\left(H_{\mathrm{pay}}\left(u\right)\right)∥N_{\mathrm{adj}}$ and share←target.4:$\left({\mathrm{idx}}_{1},\ldots ,{\mathrm{idx}}_{\kappa }\right)\leftarrow {\mathrm{Idx}}_{\kappa ,\hat{\eta }}\left(H_{\mathrm{idx}}\left(u\right)\right)$.5:**for** j=1 **to** κ **do**6:    $\mathrm{idx}\leftarrow {\mathrm{idx}}_{j}$.7:    **if** GBFPlus[idx]=0 **then**8:        **if** tag=−1 **then**9:           tag←idx.10:        **else**11:           Sample $\omega_{j}\overset{\$}{\leftarrow }{\left\{0,1\right\}}^{\lambda }$ and set $\mathrm{GBFPlus}\left[\mathrm{idx}\right]\leftarrow 1∥\omega_{j}$.12:           $\mathrm{share}\leftarrow \mathrm{share}⊕\omega_{j}$.13:        **end if**14:    **else**15:        Parse $\mathrm{GBFPlus}\left[\mathrm{idx}\right]=1∥v_{\mathrm{idx}}$ and set $\mathrm{share}\leftarrow \mathrm{share}⊕v_{\mathrm{idx}}$.16:    **end if**17:**end for**18:**if** tag=−1 **then**19:    **return** ⊥.20:**end if**21:GBFPlus[tag]←1∥share.22:c←c+1.23:**return** (GBFPlus,c).


### 5.3. Querying On-Chain Flow Attestations

RR queries the ledger directly, using the time window and traced DA identity to limit the search scope. To inspect a candidate sender ${\mathrm{DA}}_{\mathrm{se}}$ for data item *m*, RR computes the query token $u_{\mathrm{se}}^{\prime }=\hat{e}\left(s_{1}{\mathrm{KPK}}_{\mathrm{se}},\Lambda_{m}\right)$. This value matches the insertion parameter because $\hat{e}\left(k_{\mathrm{se}}{\mathrm{MPK}}_{1},\Lambda_{m}\right)=\hat{e}\left(s_{1}{\mathrm{KPK}}_{\mathrm{se}},\Lambda_{m}\right)$. RR then invokes the GBFPlus lookup in Algorithm 5.
**Algorithm 5** GBFPlus query**Input:** $\left(\kappa ,\eta ,\hat{\eta },\lambda \right)$-GBFPlus; query element $u^{\prime }\in G_{T}$; identifier length $l_{\mathrm{DA}}$; assigned identifier set N; derived hashes $H_{\mathrm{idx}},H_{\mathrm{pay}}$; index procedure ${\mathrm{Idx}}_{\kappa ,\hat{\eta }}$ **Output:** Adjacent DA identifier $N^{*}$, or failure symbol ⊥
1:Initialize $\mathrm{payload}\leftarrow 0^{\lambda }$.2:$\left({\mathrm{idx}}_{1},\ldots ,{\mathrm{idx}}_{\kappa }\right)\leftarrow {\mathrm{Idx}}_{\kappa ,\hat{\eta }}\left(H_{\mathrm{idx}}\left(u^{\prime }\right)\right)$.3:**for** j=1 **to** κ **do**4:    $\mathrm{idx}\leftarrow {\mathrm{idx}}_{j}$.5:    **if** GBFPlus[idx]=0 **then**6:        **return** ⊥.7:    **else**8:        Parse $\mathrm{GBFPlus}\left[\mathrm{idx}\right]=1∥v_{\mathrm{idx}}$ and set $\mathrm{payload}\leftarrow \mathrm{payload}⊕v_{\mathrm{idx}}$.9:    **end if**10:**end for**11:Parse $\mathrm{payload}=\mathrm{prefix}∥N^{*}$, where $|N^{*}|=l_{\mathrm{DA}}$.12:**if** $\mathrm{prefix}={\mathrm{Trunc}}_{\lambda -l_{\mathrm{DA}}}\left(H_{\mathrm{pay}}\left(u^{\prime }\right)\right)$ and $N^{*}\in N$ **then**13:    **return** $N^{*}$.14:**else**15:    **return** ⊥.16:**end if**


For honestly inserted entries queried with $u^{\prime }=u$, Algorithm 5 derives the exact κ positions. The XOR decoding recovers $\mathrm{target}={\mathrm{Trunc}}_{\lambda -l_{\mathrm{DA}}}\left(H_{\mathrm{pay}}\left(u^{\prime }\right)\right)∥N_{\mathrm{adj}}$. The prefix check binds the payload to the query element, and the suffix check confirms $N^{*}\in N$.

**Figure 4:**
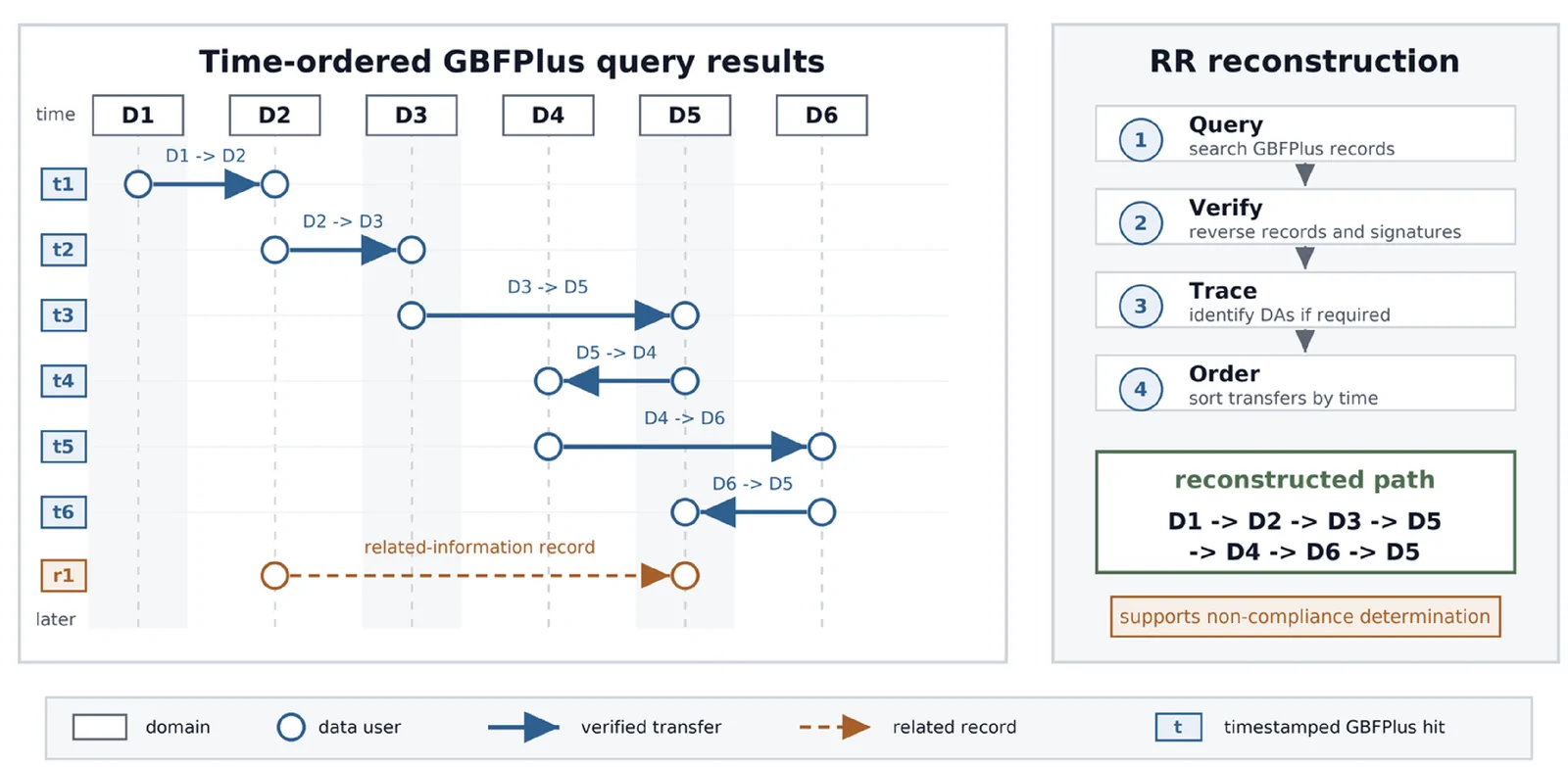
GBFPlus query outputs and candidate-path reconstruction.

If a query successfully extracts $N_{\mathrm{re}}$, RR validates the anonymous signature on the transaction block and traces its registered author via Equation (4). RR then derives $u_{\mathrm{re}}^{\prime }=\hat{e}\left(s_{1}{\mathrm{KPK}}_{\mathrm{re}},\Lambda_{m}\right)$ and scans the receiver’s incoming GBFPlus instances in the relevant time window. If this reverse lookup yields $N_{\mathrm{se}}$ and the corresponding incoming record is validly signed and traces to the receiver, RR accepts a candidate transfer edge supported by the available bilateral attestations. For *B* rotated instances in one domain and time window, the pairing-derived query element is computed once, and the filter scan costs O(Bκ) position checks. Repeating this process produces a candidate path from the committed records rather than proof that every physical transfer was logged, as shown in Figure 4.

### 5.4. Verifying On-Chain Flow Attestations

Authorized light clients (e.g., ${\mathrm{DU}}_{\mathrm{se}}$) can audit candidate paths returned by RR. For each target edge, RR samples a blinding factor $\gamma \overset{\$}{\leftarrow }Z_{q}^{*}$ and requests tokens $k_{\mathrm{se}}\gamma \Lambda_{m}$ and $k_{\mathrm{re}}\gamma \Lambda_{m}$ from the respective DAs, shielding the raw data identity during the interaction. RR checks each response via $\hat{e}\left({\mathrm{KPK}}_{i},\gamma \Lambda_{m}\right)=\hat{e}\left(Q,k_{i}\gamma \Lambda_{m}\right)$, removes the blinding factor using $\gamma^{-1}\;\mathrm{mod}\;q$, and forwards the unblinded tokens $\left(k_{\mathrm{se}}\Lambda_{m},k_{\mathrm{re}}\Lambda_{m}\right)$, transaction blocks, and Merkle authentication paths to the user.

The DU authenticates the Merkle paths against local trusted block headers and verifies transaction signatures using Algorithm 1. It then checks cross-key consistency by evaluating:   

(6) $$\begin{array}{cc} \hat{e}\left({\mathrm{KPK}}_{\mathrm{se}},\Lambda_{m}\right) & =\hat{e}\left(Q,k_{\mathrm{se}}\Lambda_{m}\right), \end{array}$$

(7) $$\begin{array}{cc} \hat{e}\left({\mathrm{KPK}}_{\mathrm{re}},\Lambda_{m}\right) & =\hat{e}\left(Q,k_{\mathrm{re}}\Lambda_{m}\right). \end{array}$$

If these pass, the user derives the query parameters:

(8) $$\begin{array}{cc} u_{\mathrm{se}} & =\hat{e}\left({\mathrm{MPK}}_{1},k_{\mathrm{se}}\Lambda_{m}\right), \end{array}$$

(9) $$\begin{array}{cc} u_{\mathrm{re}} & =\hat{e}\left({\mathrm{MPK}}_{1},k_{\mathrm{re}}\Lambda_{m}\right). \end{array}$$

The DU uses $u_{\mathrm{se}}$ and $u_{\mathrm{re}}$ as inputs to Algorithm 5 for the respective outgoing and incoming GBFPlus blocks. If the outputs resolve to $N_{\mathrm{re}}$ and $N_{\mathrm{se}}$, the DU accepts the committed bilateral evidence for the candidate edge.

To support proactive, independent retrieval, a DU must register the data item with RR before transmission and maintain full-node access. After approval, RR delivers the authorized query key $s_{1}\Lambda_{m}$, which the user validates through

(10) $$\hat{e}\left({\mathrm{MPK}}_{1},\Lambda_{m}\right)=\hat{e}\left(Q,s_{1}\Lambda_{m}\right).$$

The authorized user can subsequently evaluate $u_{j}=\hat{e}\left({\mathrm{KPK}}_{j},s_{1}\Lambda_{m}\right)$ for any candidate target domain to inspect relevant on-chain GBFPlus entries. Query authorization has an explicit lifecycle. RR uses a fresh blinding scalar for every interactive request and stops issuing new query material after authorization is revoked. Previously delivered unblinded material remains suitable for verifying immutable historical attestations. When time-scoped authorization is required, the existing hash-to-curve input can be instantiated as ${\mathrm{ID}}_{m}∥e$, where *e* is a policy epoch, yielding $\Lambda_{m,e}=H_{2}\left({\mathrm{ID}}_{m}∥e\right)$; revocation then prevents issuance for later epochs without invalidating evidence committed under an earlier epoch. All insertion, query, and verification equations remain unchanged after replacing $\Lambda_{m}$ by $\Lambda_{m,e}$.

## 6. Analysis

We first quantify GBFPlus accidental acceptance. We then prove the registration, attestation, and query-privacy results, followed by the efficiency analysis.

### 6.1. Security and Privacy Analysis

#### 6.1.1. GBFPlus Correctness and Accidental Acceptance

**Theorem 1.**  *Under the uniform κ-subset approximation induced by the deterministic distinct-index procedure ${\mathrm{Idx}}_{\kappa ,\hat{\eta }}$, and modeling $H_{\mathrm{pay}}$ as a random oracle, the probability that a query independent of the inserted elements recovers a particular λ-bit target from a $\left(\kappa ,\eta ,\hat{\eta },\lambda \right)$-GBFPlus instance is approximately*

(11) $$P_{\mathrm{GBFPlus}}\approx {\left(1-{\left(1-\frac{\kappa }{\hat{\eta }}\right)}^{\eta }\right)}^{\kappa }2^{-\lambda }.$$

**Proof.**  When a data element *u* is inserted into a $\left(\kappa ,\eta ,\hat{\eta },\lambda \right)$-GBFPlus instance, the index derivation ${\mathrm{Idx}}_{\kappa ,\hat{\eta }}\left(H_{\mathrm{idx}}\left(u\right)\right)$ is modeled as a uniformly sampled κ-subset of the position-index space $Z_{\hat{\eta }}$. For any fixed position $t\in Z_{\hat{\eta }}$, the probability that a single insertion selects *t* is $\kappa /\hat{\eta }$. After η successful insertions, the probability that position *t* remains empty is

(12) $$\mathrm{Pr}\left[\mathrm{GBFPlus}\left[t\right]=0\right]={\left(1-\frac{\kappa }{\hat{\eta }}\right)}^{\eta }.$$

For a query element $u^{\prime }$ that was not previously inserted, a structural position hit occurs when all κ mapped indices have nonzero occupancy bits. Approximating the occupancy events at distinct queried positions as independent gives ${\left(1-{\left(1-\kappa /\hat{\eta }\right)}^{\eta }\right)}^{\kappa }$; the approximation is used because insertions create weak dependence among positions. Conditioned on such a hit and on a query seed independent of the inserted seeds, the XOR result behaves as a uniformly distributed λ-bit string under the random-share and random-oracle model. It matches a particular target with probability $2^{-\lambda }$, yielding Equation (11). Unlike a conventional BF, an accidental occupancy hit is not accepted by itself: the recovered GBFPlus payload must also pass the consistency and assigned-domain checks.    □

To enforce structural data binding and route validation, the scheme parses the reconstructed payload for a query input $u^{\prime }$ as $\mathrm{prefix}∥N^{*}$ and accepts it if and only if $\mathrm{prefix}={\mathrm{Trunc}}_{\lambda -l_{\mathrm{DA}}}\left(H_{\mathrm{pay}}\left(u^{\prime }\right)\right)$ and $N^{*}\in N$. Conditioned on a structural hit, a random payload satisfies the prefix test with probability $2^{-(\lambda -l_{\mathrm{DA}})}$ and the assigned-identifier check with probability $\left|N\right|2^{-l_{\mathrm{DA}}}$. Thus, the random-acceptance probability for one directional GBFPlus block is

(13) $$P_{\mathrm{sch}}\approx {\left(1-{\left(1-\frac{\kappa }{\hat{\eta }}\right)}^{\eta }\right)}^{\kappa }\left|N\right|2^{-\lambda }.$$

Letting $P_{\mathrm{hit}}$ denote the structural hit probability:

(14) $$P_{\mathrm{hit}}\approx {\left(1-{\left(1-\frac{\kappa }{\hat{\eta }}\right)}^{\eta }\right)}^{\kappa }.$$

When the RR evaluates candidate records and the downstream receiver is not fixed a priori, the outgoing filter can return any valid adjacent identifier in N, whereas the incoming filter must return the explicit reverse upstream identifier. Under the same approximation, the random-acceptance probability across the bilateral GBFPlus check is

(15) $$P_{\mathrm{edge}}\approx P_{\mathrm{hit}}^{2}\left|N\right|2^{-2\lambda }=\frac{{\left(P_{\mathrm{sch}}\right)}^{2}}{\left|N\right|}\leq {\left(P_{\mathrm{sch}}\right)}^{2}.$$

If an audit evaluates at most *T* candidate block pairs, the union bound gives $P_{\mathrm{audit}}\leq \min\left\{1,TP_{\mathrm{edge}}\right\}$, without requiring independence across trials. The prototype value λ=32 is used to expose the performance–storage trade-off and is not presented as a 128-bit cryptographic security level. Deployments must select λ from an explicit failure budget: for target audit error ε, choose parameters satisfying $TP_{\mathrm{hit}}^{2}\left|N\right|2^{-2\lambda }\leq \varepsilon$, or conservatively increase λ to 64 or 128 bits according to the maximum number of domains, candidate blocks, and queries. This analysis follows the random-share view of GBF [38] and the distinct double-hashing implementation described by Tarkoma et al. [39].

#### 6.1.2. Registration Correctness and Key Privacy

Before analyzing the games in Section 4.3, we establish that Algorithm 2 creates a valid anonymous-attestation key and tracing record without disclosing the scalar signing key to RR. This result supplies the honest-registration relation used by the subsequent authenticity, anonymity, and traceability games.

**Theorem 2.**  *If the DLP is hard in $G_{1}$, the validated Paillier cryptosystem is IND-CPA secure, all prescribed group and ciphertext-membership checks are enforced, and RR follows Algorithm 2, then the protocol achieves key correctness, escrow-free key generation against RR, post-decryption input-consistency soundness, and tracing-record correctness. For each failed enrollment, the fresh delayed-release mask $\nu_{i}$ statistically hides the reusable master product up to distance at most 1/(q−1)*.

**Proof.**  Registration correctness and input consistency. For an honest execution of the registration protocol, ${\mathrm{DA}}_{i}$ submits group elements $X_{i}=b_{i}Q$, $Z_{i}=a_{i}P$, and the cross-product $W_{i}=a_{i}b_{i}P$. The initial pairing check executed by the RR holds due to the bilinearity of $\hat{e}$:

(16) $$\hat{e}\left(Q,W_{i}\right)=\hat{e}\left(Q,a_{i}b_{i}P\right)=\hat{e}\left(b_{i}Q,a_{i}P\right)=\hat{e}\left(X_{i},Z_{i}\right).$$

The homomorphic encryption phase over the Paillier layer evaluates:

(17) $$\begin{array}{cc} c_{i,2} & ={\mathrm{Enc}}_{{\mathrm{PuK}}_{i}}\left(\beta +\nu_{i}+\alpha_{i}b_{i}+\rho_{i}q\right),\\ \alpha_{i} & =\left(s_{2}\tau_{i}\right)\;\mathrm{mod}\;q,\;\beta =\left(s_{1}s_{2}\right)\;\mathrm{mod}\;q. \end{array}$$

Since $\alpha_{i},\beta ,\nu_{i},b_{i}<q$ and $\rho_{i}\in \left\{0,\ldots ,R_{\rho }-1\right\}$, the plaintext value avoids wrapping around the Paillier plaintext modulus M provided that $q^{2}+R_{\rho }q<M$. The bound remains sufficient because $\beta +\alpha_{i}b_{i}<q^{2}$ and $\nu_{i}+\rho_{i}q<R_{\rho }q$. Direct decryption yields ${\tilde{\mu }}_{i}=\beta +\nu_{i}+\alpha_{i}b_{i}+\rho_{i}q$. Reducing modulo *q* gives the provisional key

(18) $${\tilde{d}}_{i}={\tilde{\mu }}_{i}\;\mathrm{mod}\;q=\left(\beta +\nu_{i}+\alpha_{i}b_{i}\right)\;\mathrm{mod}\;q.$$

The integer mask $\rho_{i}q$ randomizes the Paillier plaintext representation, while the independent scalar $\nu_{i}$ is withheld until the consistency check succeeds.

For an honest input, RR derives ${\tilde{V}}_{i}=\beta Q+\alpha_{i}X_{i}+\nu_{i}Q={\tilde{d}}_{i}Q$, and the pairing check holds:

(19) $$\hat{e}\left({\tilde{V}}_{i},Z_{i}\right)=\hat{e}\left({\tilde{d}}_{i}Q,a_{i}P\right)=\hat{e}\left(a_{i}{\tilde{d}}_{i}Q,P\right)=\hat{e}\left({\tilde{A}}_{i},P\right).$$

After this check, RR releases $\nu_{i}$, and DA obtains

(20) $$d_{i}=\left({\tilde{d}}_{i}-\nu_{i}\right)\;\mathrm{mod}\;q=\left(s_{1}s_{2}+s_{2}\tau_{i}b_{i}\right)\;\mathrm{mod}\;q.$$

With $B_{i}=\tau_{i}X_{i}=\tau_{i}b_{i}Q$, the final local verification succeeds because

(21) $$\hat{e}\left(d_{i}Q,P\right)=\hat{e}\left(\left(s_{1}+\tau_{i}b_{i}\right)Q,s_{2}P\right)=\hat{e}\left({\mathrm{MPK}}_{1}+B_{i},{\mathrm{MPK}}_{2}\right),$$

confirming that the issued key aligns with the master public keys. The exceptional case $d_{i}=0$ triggers a protocol restart.

Tracing-record correctness. RR stores the static tuple $\left(T_{i}=\tau_{i}W_{i}=a_{i}\tau_{i}b_{i}P,Z_{i}=a_{i}P,{\mathrm{DA}}_{i}\right)$. Any subsequent anonymous attestation signature generated with one-time randomizer $r_{j}$ contains $R^{*}=r_{j}Q$ and $B^{*}=r_{j}B_{i}=r_{j}\tau_{i}b_{i}Q$. Evaluating RR’s tracing relation yields:

(22) $$\hat{e}\left(B^{*},Z_{i}\right)=\hat{e}\left(r_{j}\tau_{i}b_{i}Q,a_{i}P\right)=\hat{e}\left(r_{j}Q,a_{i}\tau_{i}b_{i}P\right)=\hat{e}\left(R^{*},T_{i}\right),$$

which satisfies the compliance tracing equation in Equation (4).

Soundness of the post-decryption check. Suppose that $c_{i,1}$ encrypts ${\tilde{b}}_{i}$, while $X_{i}=b_{i}Q$. The DA then obtains ${\tilde{d}}_{i}=\beta +\nu_{i}+\alpha_{i}{\tilde{b}}_{i}\;\mathrm{mod}\;q$, whereas RR constructs ${\tilde{V}}_{i}=\left(\beta +\nu_{i}+\alpha_{i}b_{i}\right)Q$. With $Z_{i}=a_{i}P$ and ${\tilde{A}}_{i}=a_{i}{\tilde{d}}_{i}Q$, Equation (19) can hold only if $a_{i}\alpha_{i}\left(b_{i}-{\tilde{b}}_{i}\right)=0\;\mathrm{mod}\;q$. Since $a_{i},\alpha_{i}\in Z_{q}^{*}$, acceptance implies $b_{i}={\tilde{b}}_{i}\;\mathrm{mod}\;q$. Otherwise, RR rejects the enrollment and does not release $\nu_{i}$. Consequently, even a failed input that encrypts zero reveals only the one-time-masked value $\beta +\nu_{i}$, rather than the reusable product β. The initial group-membership and product checks independently reject identity or malformed group inputs.

Transcript privacy and escrow freedom. For a conservative external-transcript adversary A, even granting access to all public parameters and transmitted values gives the view:

(23) $$\begin{array}{cc} \; & {\mathrm{MPK}}_{1},{\mathrm{MPK}}_{2},{\mathrm{KPK}}_{i},{\mathrm{PuK}}_{i},c_{i,1},c_{i,2},X_{i},W_{i},Z_{i},{\tilde{A}}_{i},B_{i},\nu_{i}. \end{array}$$

IND-CPA security hides the Paillier plaintext from external transcript observers. After a successful run, disclosure of the one-time mask $\nu_{i}$ does not expose $d_{i}$ because the encrypted provisional value remains hidden; after a failed run, $\nu_{i}$ is withheld. More precisely, addition of $\nu_{i}\overset{\$}{\leftarrow }Z_{q}^{*}$ maps any two candidate values of β to distributions whose statistical distance is 1/(q−1), and a fresh mask is required for every retry. RR holds $V_{i}=d_{i}Q$, $T_{i}$, and $Z_{i}$, but extracting the scalar signing key $d_{i}$ from $V_{i}$ requires solving the DLP in $G_{1}$. These arguments separately establish transcript privacy, failed-run masking, and escrow freedom; attestation unforgeability is addressed in Theorem 3 rather than being inferred from key-generation correctness.    □

#### 6.1.3. Attestation Authenticity, Anonymity, and Traceability

We next analyze $G_{\mathrm{att}-\mathrm{UF}}$, $G_{\mathrm{anon}}$, and $G_{\mathrm{trace}}$ from Section 4.3. The following theorem gives the security conclusion for accepted anonymous attestations under the stated corruption and tracing-database conditions.

**Theorem 3.**  *If the underlying BLS-type signature scheme is EUF-CMA secure [44], $H_{4}$ is modeled as a random oracle, DLP and SXDH are hard in the selected groups, and the directional co-CDH problem is hard, then every PPT adversary has negligible advantage in $G_{\mathrm{att}-\mathrm{UF}}$, $G_{\mathrm{anon}}$, and $G_{\mathrm{trace}}$. Conditional anonymity requires that $B_{i}$, $V_{i}$, RR’s tracing database, and DA signing secrets remain private and uncorrupted; traceability assumes independently generated nonzero registration scalars and an uncorrupted RR database*.

**Proof.**  $G_{\mathrm{att}-\mathrm{UF}}$. The BLS-type equation verifies possession of the ephemeral scalar corresponding to ${\mathrm{dPK}}^{*}$; EUF-CMA security prevents an external adversary from signing a fresh message under an honestly generated one-time key without that scalar. In addition, the Schnorr-type knowledge transcript [45], made non-interactive through a Fiat–Shamir challenge [46], must satisfy

(24) $$z^{*}Q=C^{*}+c^{*}R^{*},$$

where $c^{*}=H_{4}\left(\mathrm{anon}-r∥\langle M,\sigma ,{\mathrm{dPK}}^{*},Y^{*},R^{*},B^{*},C^{*}\rangle \right)$. For an honest transcript, $R^{*}=r_{j}Q$, $C^{*}=\omega_{j}Q$, and $z^{*}=\omega_{j}+c^{*}r_{j}$, so Equation (24) holds directly. By the forking argument in the random-oracle model, two accepting responses for the same commitment and distinct challenges extract $r_{j}=\left(z-z^{\prime }\right){\left(c-c^{\prime }\right)}^{-1}\;\mathrm{mod}\;q$. Hence, a PPT prover with non-negligible acceptance probability yields knowledge of the randomizer, except with the negligible forking failure probability; computing it directly from $R^{*}$ would solve DLP.

To prevent an adversary from using an arbitrary self-generated key pair, the verification equations structurally force ${\mathrm{dPK}}^{*}$ to conform to the registration framework:

(25) $$\begin{array}{cc} \hat{e}\left(Q,Y^{*}\right) & =\hat{e}\left(R^{*},{\mathrm{MPK}}_{2}\right), \end{array}$$

(26) $$\begin{array}{cc} \hat{e}\left({\mathrm{dPK}}^{*},P\right) & =\hat{e}\left({\mathrm{MPK}}_{1},Y^{*}\right)\cdot \hat{e}\left(B^{*},{\mathrm{MPK}}_{2}\right). \end{array}$$

With the extracted value *r*, Equation (25) implies $Y^{*}=r{\mathrm{MPK}}_{2}$ by non-degeneracy. Equation (26) can then be rewritten as $\hat{e}\left({\mathrm{dPK}}^{*},P\right)=\hat{e}\left(r{\mathrm{MPK}}_{1}+B^{*},{\mathrm{MPK}}_{2}\right)$. Thus, outside an honest registration, constructing the required ${\mathrm{dPK}}^{*}=s_{2}\left(r{\mathrm{MPK}}_{1}+B^{*}\right)\in G_{1}$ for a nonzero self-chosen base is exactly the $G_{2}$-to-$G_{1}$ co-CDH direction, while the BLS equation additionally binds the resulting one-time key to *M*. An accepting fresh tuple produced with non-negligible probability would therefore contradict either the extractor, directional co-CDH, or BLS EUF-CMA assumption.

$G_{\mathrm{anon}}$. Each signature uses a fresh $r_{j}$ to form $R^{*}=r_{j}Q$, $B^{*}=r_{j}B_{i}$, ${\mathrm{dPK}}^{*}=r_{j}V_{i}$, and $Y^{*}=r_{j}{\mathrm{MPK}}_{2}$, while the response transcript uses an independent commitment $C^{*}=\omega_{j}Q$. A hybrid replaces the common-scalar relations involving the unpublished bases $B_{i}$ and $V_{i}$ with random group elements. Distinguishing adjacent hybrids gives a DDH distinguisher in one of the source groups and therefore contradicts SXDH. In the final hybrid the challenge distributions for $i_{0}$ and $i_{1}$ are identical, so ${\mathrm{Adv}}_{A}^{\mathrm{anon}}$ is negligible, assuming that the two signing secrets, registration values, and RR’s private tracing state remain confidential.

$G_{\mathrm{trace}}$. For an honest signature from ${\mathrm{DA}}_{i}$, expanding Equation (4) using RR’s private tracing record yields $\hat{e}\left(B^{*},Z_{i}\right)=\hat{e}\left(r_{j}\tau_{i}b_{i}Q,a_{i}P\right)=\hat{e}\left(r_{j}Q,a_{i}\tau_{i}b_{i}P\right)=\hat{e}\left(R^{*},T_{i}\right)$. The knowledge extraction and registration equations used in the unforgeability argument prevent an accepted tuple from escaping the registered relation without solving DLP or directional co-CDH. For two independently generated honest records, a false match requires equality of the nonzero tag scalars $\tau_{i}b_{i}=\tau_{h}b_{h}$, which occurs with probability 1/(q−1). For a database of *n* records, the probability that one honest signature matches any incorrect record is at most (n−1)/(q−1) by the union bound. Thus, ${\mathrm{Adv}}_{A}^{\mathrm{trace}}$ is negligible when the tracing database is intact.    □

#### 6.1.4. Flow-Metadata and Query Privacy

It remains to analyze $G_{\mathrm{qpriv}}$. The theorem below covers both the public ledger view and repeated interactive requests to a processing DA, while making the unavoidable public metadata and prior-token conditions explicit.

**Theorem 4.**  *If the co-CDH problem is hard and the domain-separated outputs of $H_{1}$ are modeled as random oracles, then every PPT adversary has negligible advantage in $G_{\mathrm{qpriv}}$, conditioned on equal public ledger metadata and the absence of a previously disclosed unblinded token for either challenge identifier. The claim is subject to the stated occupancy/rotation leakage and to the per-attempt random-acceptance bounds $P_{\mathrm{sch}}$ and $P_{\mathrm{rand}}$*.

**Proof.**  Ledger-view hybrid. The public values relevant to an external observer are ${\mathrm{MPK}}_{1}=s_{1}Q$, ${\mathrm{KPK}}_{i}=k_{i}Q$, and, when available in an authorized interaction, the group-encoded identifier $\Lambda_{m}$. Under the co-CDH assumption, the observer cannot derive $k_{i}\Lambda_{m}$ from ${\mathrm{KPK}}_{i}$ and $\Lambda_{m}$, nor $s_{1}\Lambda_{m}$ from ${\mathrm{MPK}}_{1}$ and $\Lambda_{m}$. It therefore cannot construct $u=\hat{e}\left(s_{1}k_{i}Q,\Lambda_{m}\right)=\hat{e}\left({\mathrm{MPK}}_{1},k_{i}\Lambda_{m}\right)=\hat{e}\left({\mathrm{KPK}}_{i},s_{1}\Lambda_{m}\right)$. In the random-oracle hybrid, replacing the unknown value *u* by a fresh target makes $H_{\mathrm{idx}}\left(u\right)$ and $H_{\mathrm{pay}}\left(u\right)$ independent of the challenge bit. Distinguishing this replacement yields a directional co-CDH solver or requires querying the oracle at the hidden target. Adaptive nonchallenge queries do not reveal that target because the hashes are domain separated and the challenge identifiers have no disclosed unblinded token.

A guessed query seed is accepted with probability approximately $P_{\mathrm{sch}}$ from Equation (13). If A instead selects occupied cells directly and XORs an arbitrary κ-tuple, then, under the random-share model, the conditional probability that the reconstructed string passes both payload tests is

(27) $$P_{\mathrm{rand}}=\left|N\right|2^{-l_{\mathrm{DA}}}2^{-(\lambda -l_{\mathrm{DA}})}=\left|N\right|2^{-\lambda }.$$

For at most $Q_{g}$ independent guessed seeds or arbitrary reconstructions, the corresponding success probability is bounded by $\min\{1,Q_{g}P_{\mathrm{sch}}\}$ or $\min\{1,Q_{g}P_{\mathrm{rand}}\}$, respectively. Accordingly, GBFPlus provides stronger metadata protection than a conventional public BF index: the latter exposes a stable public membership index, whereas a GBFPlus observer lacks both the pairing-derived positions and the payload-consistency value. The construction nevertheless reveals transaction timing, filter dimensions, occupancy, direction, and the public filter-rotation sequence; it does not provide zero leakage or hide access patterns revealed outside the ledger.

Interactive-query hybrid. RR samples a fresh $\gamma \overset{\$}{\leftarrow }Z_{q}^{*}$ for each request and sends $\gamma \Lambda_{m}$. For every fixed nonidentity $\Lambda_{m}$, multiplication by uniform γ is uniform over $G_{2}\setminus \left\{0_{G_{2}}\right\}$, so the blinded element is information-theoretically independent of the identifier in each request. A sequence of adaptive challenge interactions remains independent because every request uses an independent γ. Combining the ledger and interactive hybrids gives negligible ${\mathrm{Adv}}_{A}^{\mathrm{qpriv}}$ under the challenge conditions defined above.

An unblinded authorization token is intentionally usable for deterministic verification and therefore lies outside the indistinguishability challenge once disclosed. Revocation stops future issuance but does not erase the verification capability of material already delivered for immutable historical records. The epoch-scoped instantiation $\Lambda_{m,e}$ described above limits new authorization to approved policy epochs while preserving earlier evidence.

Finally, ledger persistence makes retrospective modification of a committed GBFPlus transaction detectable, and SPV paths allow a light client to verify transaction inclusion. These guarantees apply to submitted attestations and do not establish the completeness or factual occurrence of events outside the submitted records.    □

#### 6.1.5. Security Conclusion

The registration theorem establishes the honest key and tracing relations required by the games. Theorem 3 then gives negligible advantage in attestation forgery, signer identification, and trace evasion, satisfying Integrity and Authenticity, Conditional Anonymity, and Traceability. Theorem 4 gives negligible distinguishing advantage for equal-metadata ledger views and freshly blinded interactive requests, satisfying Query Confidentiality subject to the stated leakage and authorization conditions. Together with ledger persistence and inclusion verification, these results establish the security requirements in Section 4.3 for committed attestation evidence.

### 6.2. Efficiency Analysis

For fixed κ, BF, GBF, and GBFPlus have constant expected per-record filter cost; when κ is explicit, insertion and query are O(κ), while scanning *B* rotated filters is O(Bκ). A BF stores one public membership bit per position, whereas a GBF stores a λ-bit XOR share at every position. GBFPlus retains the GBF share mechanism, adds one occupancy bit per position, and stores payloads only at occupied positions. Its privacy advantage is therefore not lower asymptotic cost: it is the absence of a stable public data-hash index, plus controlled recovery of an adjacent-domain identifier only after the consistency-prefix and assigned-domain checks.

Let $T_{\mathrm{idx}}$ be the expected cost of ${\mathrm{Idx}}_{\kappa ,\hat{\eta }}$, including position hashes, duplicate suppression, and retries; $T_{r}$ the cost of sampling one random λ-bit share; $T_{⊕}$ one λ-bit XOR; $T_{H}$ one domain-separated $H_{1}$ evaluation; and $T_{\mathrm{val}}$ the occupancy, parsing, prefix, and assigned-domain checks in a lookup. Define $T_{\mathrm{tok}}=T_{\mathrm{sm}}+T_{bp}+T_{\mathrm{ser}}$ as the scalar-multiplication, pairing, and target-group serialization cost needed to derive *u* or $u^{\prime }$. Table 2 reports dominant per-record costs; ordinary cell-access costs are common and omitted. If *a* is the actual number of occupied cells, sparse GBFPlus storage is exactly $\hat{\eta }+a\lambda$ bits, where $a\leq \min\{\hat{\eta },\kappa \eta \}$. The $T_{\mathrm{tok}}$ term corresponds to experimental Stage 1, while all remaining filter terms correspond to Stage 2; when a query token is reused across *B* rotated filters, $T_{\mathrm{tok}}$ is paid once rather than *B* times.

### 6.3. Experimental Setup and Results

Empirical evaluations were conducted on a Windows 11 system equipped with 32 GB of RAM, an Intel Core Ultra X7 358H processor, and Go 1.26.2. Curve and pairing operations use the bls12-381 package (https://github.com/kilic/bls12-381, accessed on 29 June 2026), while the enrollment test uses a math/big-based Paillier implementation with a 2048-bit modulus. Both the long-term transfer confirmation under $k_{i}$ and the anonymous on-chain attestation under $d_{i}$ use BLS-type signatures [44]. The prototype parameters are κ=12, λ=32, and $l_{\mathrm{DA}}=20$. Both the theoretical storage analysis in Table 3 and the measured latency workloads in Table 4 cover η≤512. The value λ=32 is an experimental setting for measuring storage and latency. A deployment requiring a lower accidental-acceptance probability can select a larger payload, such as 64 or 128 bits, at a proportional payload-storage cost. The position mappings use double hashing [39]:

(28) $$H_{i}\left(x\right)=\left(H^{\prime }\left(x\right)+\left(i-1\right)H^{\prime \prime }\left(x\right)\right)\;\mathrm{mod}\;\hat{\eta },\;i=1,\ldots ,\kappa ,$$

where $H^{\prime }$ uses SHA-256 and $H^{\prime \prime }$ uses MurmurHash3. This implementation approximates the uniform κ-subset model used in the analysis because duplicate positions are discarded.

Table 3 instantiates the sparse bound $\hat{\eta }+\lambda \min\left\{\hat{\eta },\kappa \eta \right\}$ from Table 2 at κ=12 and λ=32. For example, the η=512 row gives 8864+32×(12×512)=205,472 bits. Because records can share positions, the physical payload count is normally smaller; the table deliberately reports a capacity-planning ceiling rather than measured heap use.

The implementation of ${\mathrm{Idx}}_{\kappa ,\hat{\eta }}$ evaluates Equation (28) on x∥ctr with an incrementing counter and discards duplicate positions until it obtains κ distinct indices. Each GBFPlus operation follows the decomposition in Table 2: Stage 1 measures $T_{\mathrm{tok}}$, namely scalar multiplication, one pairing, and target-group serialization, while Stage 2 measures the two domain-separated hashes, $T_{\mathrm{idx}}$, share processing, occupancy access, and query validation. The hash-to-curve computation of the already specified input $\Lambda_{m}$ is performed before this timed interval and is therefore outside the reported scope. Stage 2 values are averaged across 40 independently initialized filters. Runtime garbage collection is completed before each timed section and suspended within that section so that a collection pause is not assigned to a particular batch size. The two extended workloads, η=448 and 512, are launched first in a fresh process to keep their timing conditions independent of preceding batches. In Table 4, “Insert (ms, total)” and “Query (ms, total)” are the aggregate Stage 1 token-derivation times for all η insertion and query tokens, rather than end-to-end operation totals. “Stage 1 (ms/op)” normalizes the two aggregate derivation times, while the final insertion and query columns report Stage 2 filter processing per operation. A one-filter operation is therefore approximated by the corresponding per-operation Stage 1 and Stage 2 sum.

Across the seven batch sizes, Stage 1 remains between 0.4756 and 0.5085 ms per operation, whereas Stage 2 insertion and query remain below 2.2 μs per operation. Hence, $T_{\mathrm{tok}}$ is more than two orders of magnitude larger than the measured filter-only work and dominates a one-filter query. For a fixed data item and domain, however, the derived query element can be reused across relevant rotated filters: the scan pays Stage 1 once and then repeats only the domain-separated hashes, distinct-index derivation, occupancy/XOR processing, prefix comparison, and assigned-domain check represented by the non-token terms in Table 2.

**Figure 5:**
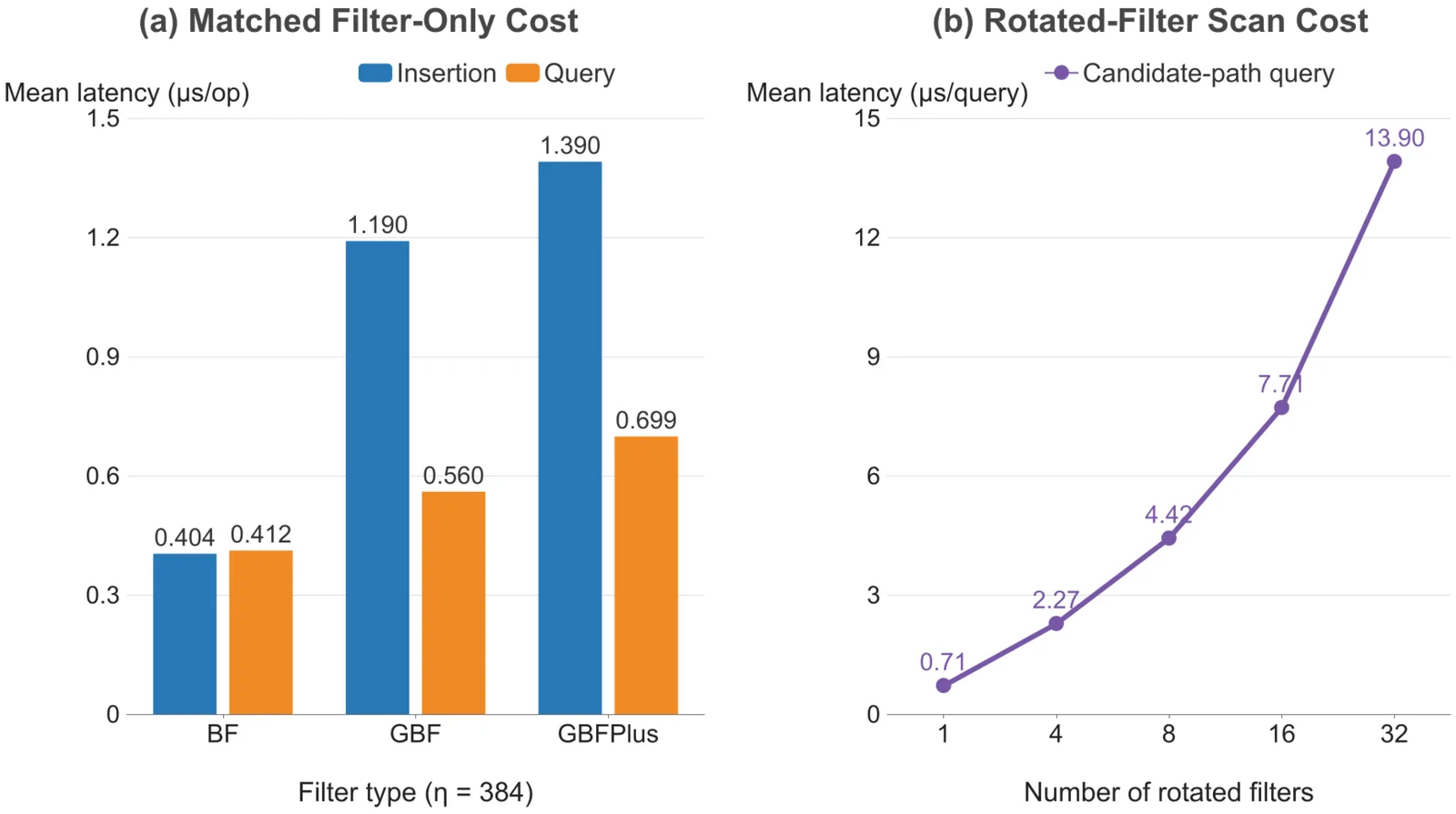
Matched filter-only measurements with pairing-derived Stage 1 excluded: (**a**) BF, GBF, and GBFPlus insertion and successful-query latency at η=384; (**b**) GBFPlus candidate-path latency with the match in the last rotated filter. Numeric labels are means.

The matched filter-only comparison uses κ=12, $\hat{\eta }=6648$, η=384, and a 32-bit payload for GBF and GBFPlus. Six timing chunks each process 30 independently initialized filters, giving 69,120 insertions and 69,120 successful queries per filter type. Pairing-based token derivation is excluded from all Figure 5a values because it is isolated as Stage 1 in Table 4. The blue insertion bars report 0.404, 1.190, and 1.390 μs for BF, GBF, and GBFPlus; the orange query bars report 0.412, 0.560, and 0.699 μs. Thus, GBFPlus costs 1.17× the GBF insertion latency and 1.25× the GBF query latency, consistent with the explicit occupancy and payload-validation work in Table 2; the comparison does not claim a speed advantage over BF.

Candidate-path lookup was also measured with the matching entry placed in the last of 1, 4, 8, 16, or 32 rotated filters. Each labeled point in Figure 5b comprises 200,000 queries and excludes the reusable Stage 1 token. Mean filter-only scan time rises monotonically from 0.711 μs for one filter to 13.899 μs for 32 filters, matching the O(Bκ) scan term stated above; Stage 1 must be added once for an end-to-end path query. In 40 load trials at each of 192, 288, and 384 target entries, no collision-induced rotation was observed among 34,560 total insertion attempts. This finite experiment does not imply zero conflict probability, so production implementations must retain the rotation procedure. A separate negative test produced no accepted result among 100,000 random queries against a populated filter. This observation is consistent with, but does not estimate, Equation (13): the 12-bit prefix alone matches with probability $2^{-12}\approx 0.024\%$, while complete acceptance additionally requires the structural hit and a valid assigned-domain suffix.

The traceable anonymous attestation signature ς contains the BLS signature σ, the ephemeral key ${\mathrm{dPK}}^{*}$, the registration tuple $\left(Y^{*},R^{*},B^{*}\right)$, and the transcript $\left(C^{*},z^{*}\right)$. The benchmark implements the complete revised protocol: anonymous signing includes commitment generation, the context-bound $H_{4}$ challenge, and response computation, while verification includes Equation (24) and all three pairing-based relations in Algorithm 1. Table 5 reports arithmetic means, medians, and population standard deviations. The enrollment row uses a 2048-bit Paillier modulus, and the trace row scans 64 records with the correct record placed last.

All 120 independently generated valid anonymous signatures were accepted. The negative suite rejected all 240 modified-message or modified-response cases, and tracing selected the correct registration record while rejecting an unrelated record. Together with the unit tests for Paillier homomorphism, enrollment relations, BLS message binding, bilinear token equality, GBFPlus round-trip recovery, and atomic conflict handling, these results verify functional consistency of the implemented algorithms. They do not constitute a cryptographic security proof or an end-to-end blockchain evaluation.

## 7. Discussion and Limitations

The framework protects the content and cryptographic origin of committed attestations rather than guaranteeing that every physical transfer is logged. Under the stated model, registered DAs follow the submission protocol. If an operational omission is nevertheless suspected after a dispute, the counterparty-held transfer signature can be compared with the outgoing and incoming ledger records: a valid signed receipt without a corresponding filter attestation provides evidence for post-hoc investigation and attribution. This is an organizational audit mechanism, not a cryptographic solution to bilateral collusion; if both parties suppress all evidence, the scheme cannot reconstruct the omitted event.

The anonymous attestation scheme hides the signing DA’s cryptographic identity from ordinary ledger observers while preserving RR’s tracing authority. RR therefore remains trusted for query authorization, tracing-record confidentiality, and availability.

Compared with the conventional BF traceability design of Zhao et al. [22], the primary advantage is privacy rather than a universal performance improvement. Their public and alliance-chain workflow uses a stable data-hash index together with explicit domain, blockchain, and user identifiers to support authorization and traceability. GBFPlus instead derives hidden positions from data-specific pairing material, constrains the recovered payload, aggregates multiple events, and combines the filter with an anonymous yet traceable upload signature. Consequently, ordinary ledger observers do not receive a directly queryable data identifier, adjacent-domain relation, or uploader identity. This added privacy entails a pairing and payload-storage overhead that a conventional BF does not incur.

## 8. Conclusions

This paper presents a privacy-preserving on-chain attestation framework for recorded cross-domain data flows. By combining GBFPlus, bilateral attestations, ledger verification, and accountable anonymous signatures, the framework supports auditable reconstruction of committed transfer evidence while reducing the exposure of sensitive flow metadata. The security and efficiency analyses characterize the principal guarantees and costs of the construction under the stated system model, and the prototype results demonstrate the functional feasibility of the evaluated filter, signing, verification, and tracing procedures.

The framework is intended for permissioned regulatory environments in which registered domain gateways preserve attestations of observed transfers and an authorized regulator conducts later audits. Its guarantees apply to evidence committed by participating gateways: bilateral records and signatures strengthen later verification and attribution, while the ledger makes retrospective modification detectable. Cryptographic signer anonymity protects the uploader’s identity from ordinary ledger observers.

## Figures and Tables

**Table 1:** Core functional and privacy comparison.

Approach	Query or Evidence	Storage Form	Accidental Result/Insertion	Ledger-Visible Information
BF	Public membership result	$\hat{\eta }$ bits	No insertion failure; occupancy false positives	Stable public index and occupancy
GBF	Recovery of a λ-bit value	$\hat{\eta }\lambda$ bits	May fail when all selected cells are set; random recovery $2^{-\lambda }$	Share array; privacy depends on query-position secrecy
Zhao et al. [22]	Cross-domain authorization and graph tracing	BF plus explicit identifiers	Conventional BF behavior	Stable data-hash, domain, blockchain, and user identifiers
General ZK proof	Verification of a circuit statement	Proof and public inputs	Soundness error; no filter-insertion process	Public inputs, proof, and transaction metadata
GBFPlus	Authorized candidate-edge recovery and committed evidence	$\hat{\eta }+a\lambda$ bits, $a\leq \min\{\hat{\eta },\kappa \eta \}$	Rotate and retry on insertion failure; acceptance bounded by $P_{\mathrm{sch}}$ and $P_{\mathrm{edge}}$	Dimensions, occupancy, direction, and rotation; target, adjacent domain, and uploader identity remain cryptographically protected

**Table 2:** Per-record computation cost and storage bounds.

Filter Type	Insertion Complexity	Query Complexity	Storage Bound (bits)
BF	$T_{\mathrm{idx}}$	$T_{\mathrm{idx}}$	$\hat{\eta }$
GBF	$T_{\mathrm{idx}}+\left(\kappa -1\right)\left(T_{r}+T_{⊕}\right)$	$T_{\mathrm{idx}}+\kappa T_{⊕}$	$\hat{\eta }\lambda$
GBFPlus	$T_{\mathrm{tok}}+2T_{H}+T_{\mathrm{idx}}+\left(\kappa -1\right)\left(T_{r}+T_{⊕}\right)$	$T_{\mathrm{tok}}+2T_{H}+T_{\mathrm{idx}}+\kappa T_{⊕}+T_{\mathrm{val}}$	$\hat{\eta }+\lambda \min\left\{\hat{\eta },\kappa \eta \right\}$

**Table 3:** Theoretical GBFPlus storage upper bounds.

Maximum Insertions (η)	128	192	256	320	384	448	512
Number of GBFPlus Positions $\left(\hat{\eta }\right)$	2216	3324	4432	5540	6648	7756	8864
Storage Upper Bound (bits)	51,368	77,052	102,736	128,420	154,104	179,788	205,472

**Table 4:** Measured GBFPlus latency.

η	$\hat{\eta }$	Insert (ms, Total)	Query (ms, Total)	Stage 1 (ms/op)	Insert (μs/op)	Query (μs/op)
128	2216	61.394	60.369	0.4756	1.870	1.125
192	3324	92.712	92.052	0.4812	1.673	1.111
256	4432	128.346	120.542	0.4861	1.462	1.492
320	5540	152.205	154.655	0.4795	1.638	1.042
384	6648	185.222	180.331	0.4760	1.742	1.034
448	7756	222.202	222.278	0.4961	1.834	1.001
512	8864	263.253	257.471	0.5085	2.134	0.982

**Table 5:** Measured Go Cryptographic Operation Latency.

Operation	Samples	Mean (ms)
Validated enrollment (2048-bit Paillier)	10	95.679
Long-term BLS signing	768	0.311
Long-term BLS verification	768	0.746
Anonymous signing	192	0.731
Anonymous verification	192	2.086
Trace scan over 64 records (match last)	12	34.630

## Data Availability

Data are contained within the article.
